# An albumin-based tumor-targeted oxaliplatin prodrug with distinctly improved anticancer activity *in vivo* †
†Electronic supplementary information (ESI) available: Experimental materials and methods, characterization details of all compounds, X-ray crystal structures. CCDC 1499561 and 1499562. For ESI and crystallographic data in CIF or other electronic format see DOI: 10.1039/c6sc03862j



**DOI:** 10.1039/c6sc03862j

**Published:** 2016-12-15

**Authors:** Josef Mayr, Petra Heffeter, Diana Groza, Luis Galvez, Gunda Koellensperger, Alexander Roller, Beatrix Alte, Melanie Haider, Walter Berger, Christian R. Kowol, Bernhard K. Keppler

**Affiliations:** a University of Vienna , Institute of Inorganic Chemistry , Waehringer Strasse 42 , A-1090 , Vienna , Austria . Email: christian.kowol@univie.ac.at ; Fax: +43-1-4277-52680 ; Tel: +43-1-4277-52609; b Institute of Cancer Research and Comprehensive Cancer Center , Medical University of Vienna , Borschkegasse 8a , A-1090 , Vienna , Austria . Email: petra.heffeter@meduniwien.ac.at ; Fax: +43-1-40160-957555 ; Tel: +43-1-40160-57557; c Research Platform “Translational Cancer Therapy Research” , University of Vienna , Waehringer Strasse 42 , A-1090 , Vienna , Austria; d University of Vienna , Institute of Analytical Chemistry , Waehringer Strasse 38 , A-1090 , Vienna , Austria

## Abstract

The design of targeted platinum(iv) prodrugs is a very promising approach to enhance the low selectivity of platinum(ii) drugs towards cancerous tissue in order to reduce the impact on healthy tissue and, consequently, the often severe side-effects. Herein, we report a set of mono-functionalized cis- and oxaliplatin-based platinum(iv) complexes bearing a maleimide moiety, which allows selective binding to serum albumin in the bloodstream. This leads not only to a prolonged plasma half-life by avoidance of fast renal clearance, but also to preferential accumulation of the drug in the tumor tissue due to the EPR-effect. Additionally, analogous succinimide-functionalized derivatives were prepared to verify the influence of the maleimide moiety. First experiments showed that all the maleimide compounds are stable and also possess good albumin-binding properties in whole serum. Further analytical studies on *in vivo* samples proved the highly increased plasma half-life, as well as tumor accumulation of the maleimide-functionalized substances. *In vivo* antitumor experiments with CT-26-bearing mice showed that, in contrast to the cisplatin derivatives, the oxaliplatin-based complexes had exceptionally better activity than the free drug resulting in the cure of the majority of treated mice. Subsequent analysis suggested that a distinctly faster reduction as well as reduced tumor accumulation of the cisplatin derivative might explain the worse performance compared to the oxaliplatin(iv) complexes. Taken together, a novel lead platinum(iv) complex with outstanding antitumor activity is presented, which will now be further developed towards clinical phase I trials.

## Abstract 2

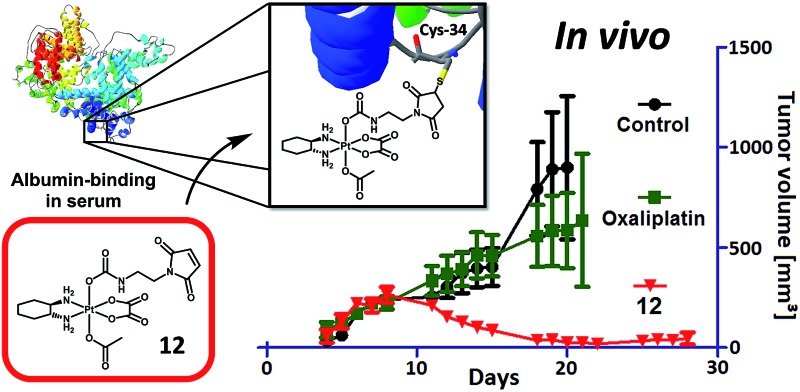
An oxaliplatin-based platinum(iv) drug which specifically binds to albumin after i.v. application led to several complete responses in tumor-bearing mice.

## Introduction

Since the discovery of the anticancer activity of cisplatin by Rosenberg *et al.* in the 1960s,^1^ and its approval in 1978, the success of this platinum(ii)-based complex has not been stopped.^2^ Cisplatin is still one of the most frequently used chemotherapeutics *e.g.* against lung, head and neck, testicular, ovarian, and bladder cancer. However, the treatment is frequently accompanied by severe side-effects and acquired or intrinsic resistance.^3^ Aside of cisplatin, only two additional platinum(ii) complexes have been approved worldwide, namely carboplatin and oxaliplatin. While carboplatin is similar to cisplatin in its area of application, oxaliplatin is especially active against colon carcinoma.^4^ Nevertheless, the low selectivity of platinum(ii) drugs towards cancer cells and cancerous tissue, together with severe adverse effects are still major disadvantages.^5,6^ An approach to enhance the selectivity are platinum(iv) prodrugs, which are chemically more inert and thus less cytotoxic. After entering the hypoxic, reductive conditions of the cancerous tissue, they are reduced and release their active, highly cytotoxic Pt(ii)-core (activation by reduction).^7^ Consequently, the adverse effects on healthy tissue should be reduced. From a chemical point of view, the two additional ligands of octahedral platinum(iv) compounds offer a broad range of possibilities to modify such compounds and to fine-tune their pharmacological properties like lipophilicity or solubility.^8^ Nevertheless, only a few of a countless number of platinum(iv) compounds prepared ended in clinical trials, with satraplatin as the most prominent representative. Satraplatin reached a clinical phase III study in combination with prednisone, called SPARC (satraplatin and prednisone against hormone-refractory prostate cancer) trial. However, this platinum(iv) drug failed to show convincing benefits compared to standard therapy and therefore satraplatin was not approved for cancer therapy.^9^ Notably, some studies showed that satraplatin is already unselectively reduced in blood and/or with liver enzymes to the platinum(ii) complex.^10,11^

In general, there are several possibilities to improve the selectivity of platinum(iv) compounds. An option would be to exploit tumor-specific properties (*e.g.* overexpression of diverse receptors) *e.g.* by using targeting molecules like vitamins or peptides, a strategy known as “active targeting”. However, as such drugs are usually below a size of 60 kDa, they are still susceptive to renal clearance.^12^ On the other hand, tumor-specific activity can also be achieved by “passive targeting” strategies *via* the so-called enhanced permeability and retention (EPR) effect.^13^ This effect can be described by the combination of fenestrated blood vessels together with a lack of a lymph drainage system in the solid tumor tissue, which results in tumor-specific accumulation of macromolecules like nanoparticles and large proteins.^14–16^ One of the most elegant and promising ways to exploit the EPR effect is the use of human serum albumin (HSA) as a carrier.^17,18^ Abraxane®,^19,20^ an albumin-bound nanoparticle formulation of paclitaxel, which was approved for metastatic breast cancer, non-small cell lung cancer (NSCLC) and pancreas adenocarcinoma, as well as the doxorubicin-based drug aldoxorubicin,^21^ which is currently in clinical phase III studies, are two successful examples. Aldoxorubicin possesses a maleimide moiety, which is known to rapidly bind to the free thiol group (cysteine at position 34)^22^ of albumin exploiting it as a carrier without the use of exogenous compounds usually used for nanoformulations. With regard to platinum compounds, there are a few publications of platinum(ii) complexes, which possess a maleimide moiety attached to the leaving group.^23,24^ The drawback of coupling to a platinum(ii) compound is that in this oxidation state the compounds are quite reactive, especially considering the long plasma half-life of albumin. In addition, a platinum(iv) complex with a highly hydrophobic C_16_ alkyl chain with non-covalent albumin binding properties has been reported, however, with the disadvantage of low aqueous solubility.^25^ We recently presented a proof-of-principle for bis-maleimide-functionalized platinum(iv) complexes.^26^ However, albumin-binding studies indicated that despite the presence of two maleimide moieties, only one albumin molecule is able to bind these prodrugs.^26^ Thus, the second maleimide is free to (unspecifically) react with other proteins or thiol-containing molecules, which might lead to an uncontrolled pharmacological behavior *in vivo*. Therefore, we herein report on the first mono-maleimide platinum(iv) complexes not only of oxaliplatin but also of cisplatin. Unexpectedly, only the oxaliplatin derivatives exerted distinctly enhanced activity as compared to the free drug, while the cisplatin prodrugs failed to result in improved anticancer effects *in vivo*. Moreover, we discovered that small structural modifications on one axial ligand resulted in distinct differences in the anticancer activity of the novel compounds. This finally led to the identification of a highly *in vivo*-active oxaliplatin-releasing derivative, which is now a lead candidate for further preclinical evaluations.

## Results and discussion

**Scheme 1:**
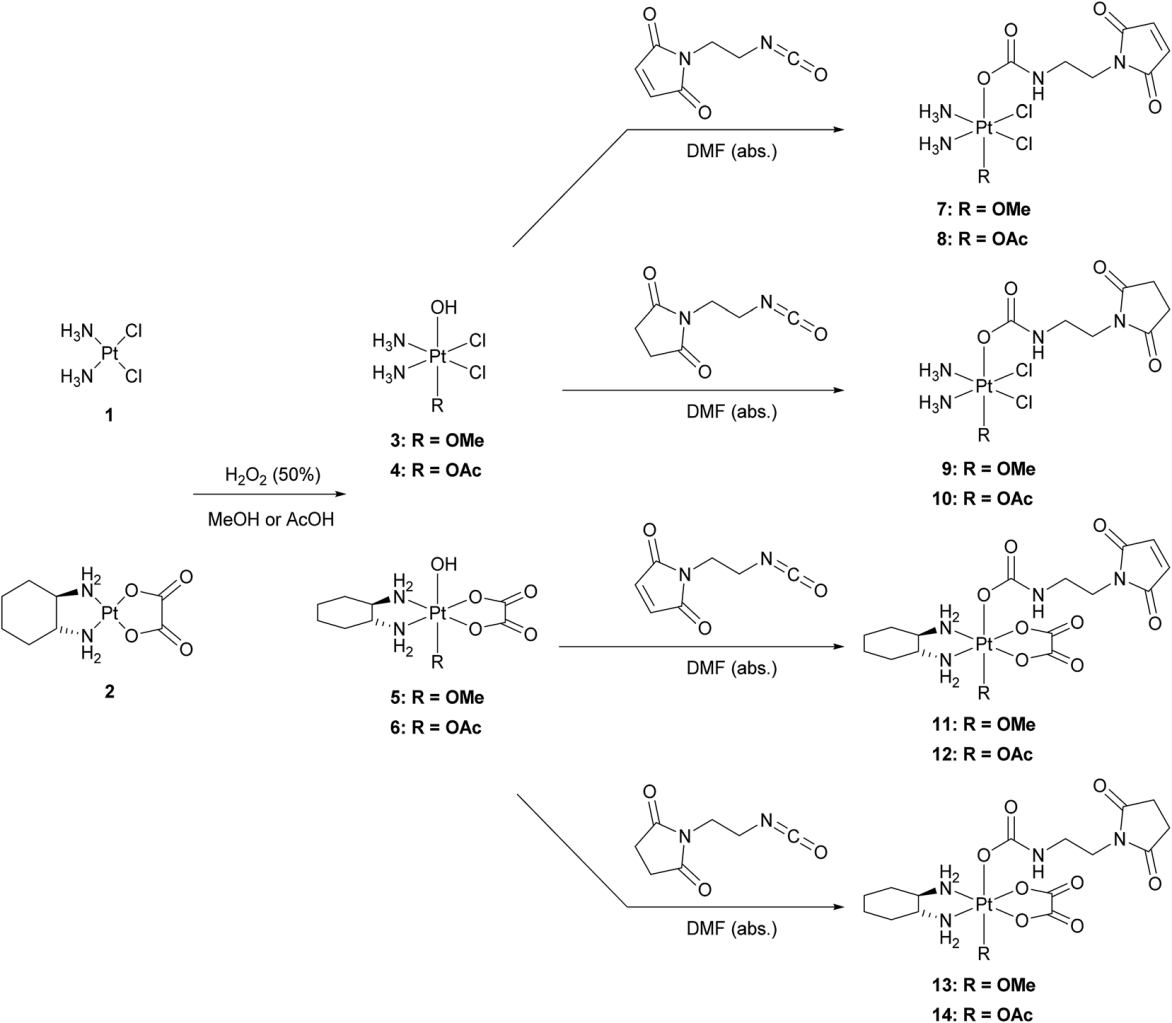
Reaction scheme for the synthesis of the mono-functionalized platinum(iv) complexes **7–14**.

The platinum(ii) precursors for the syntheses of the novel platinum(iv) prodrugs were cisplatin (**1**) or oxaliplatin (**2**). In the first step, the platinum(ii) compounds were oxidized with hydrogen peroxide (50%) using either methanol^27^ or acetic acid^28^ as the solvent, to yield the unsymmetrically oxidized platinum(iv) precursors **3–6**. The maleimide- and succinimide-functionalized ligands were prepared by conversion of the propionic acids to the corresponding isocyanates.^26^ Finally, all eight mono-functionalized complexes were prepared *via* reaction of the isocyanate-containing ligands and the hydroxide function of the platinum(iv) precursors (Scheme 1). All novel compounds were purified *via* preparative RP-HPLC with moderate yields between 18–43% and characterized by high resolution mass spectrometry, NMR spectroscopy and elemental analysis (see ESI†).

### Single-crystal X-ray diffraction analysis

**Fig. 1:**
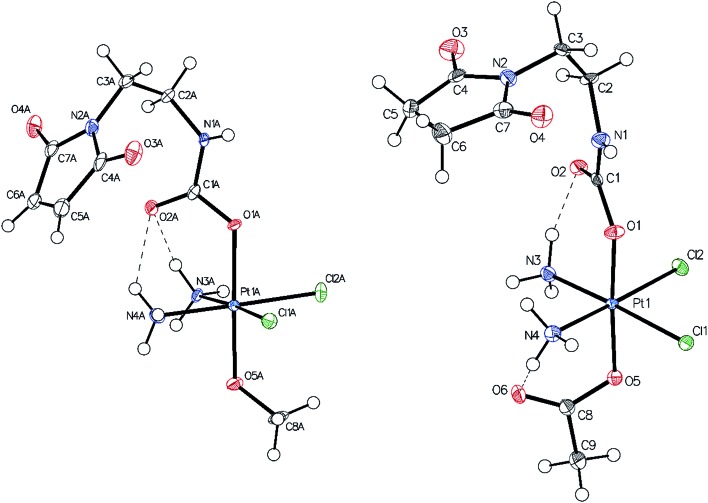
ORTEP plot of the single crystal X-ray diffraction analysis of **7** (left) and **10** (right). The thermal ellipsoids have been drawn at the 50% probability level (the second molecule of the asymmetric unit of **7** and the water molecule of the asymmetric unit of **10** were omitted for clarity).

Single-crystals of compounds **7** and **10** (CCDC ; 1499561 and ; 1499562†) suitable for X-ray diffraction analysis were obtained out of saturated solutions of the compounds in H_2_O/MeOH/Et_2_O mixtures. Compound **7** crystallized in the monoclinic space group *P*2_1_/*c* (Fig. 1) with two molecules in the asymmetric unit, which differ in the orientation of the maleimide-bearing axial ligand and their corresponding hydrogen bonds. Complex **10** co-crystallized with one water molecule in the monoclinic space group *C*2/*c* (Fig. 1). Both compounds are cisplatin-derivatives, with two ammine and two chlorido ligands in the equatorial plane bound to the platinum center. The ligands show typical binding lengths of around 2.04 Å, in the case of the Pt–N, and approximately 2.32 Å for the corresponding Pt–Cl bonds.^29^ Also the Pt–O binding length of about 1.98 Å in the case of the methoxido ligand is comparable to Pt(N(CH_3_)_2_CH_2_CH_2_NH_2_)Cl_2_(OH)(OMe)^30^ and the Pt–O distance of the acetato ligand with ∼2.01 Å is similar to Pt(NH_3_)_2_Cl_2_(OAc)_2_ (see ESI†).^31^

### Stability and albumin-binding studies

**Fig. 2:**
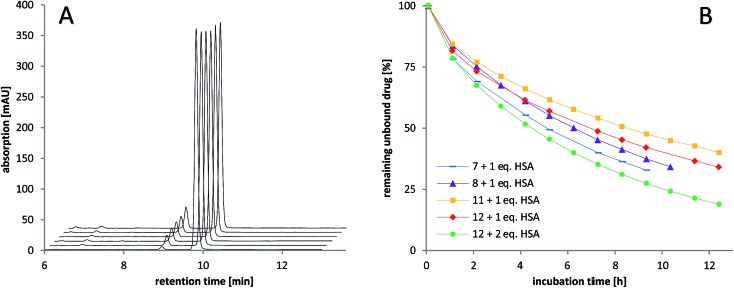
RP-HPLC studies of (A) the stability of the maleimide-oxaliplatin(iv) compound **11** in aqueous solution after mixing (10 min), 1 h, 2 h, 3 h, 4 h, and 10 h (front to back) and (B) the albumin-binding rate of the maleimide-containing compounds **7–8** (cisplatin core) and **11–12** (oxaliplatin core).

The stability of the compounds in aqueous solution was monitored over several hours using analytical RP-HPLC. All of the maleimide-containing compounds **7–8** and **11–12** showed slow hydrolysis with less than 1% h^–1^ (see Fig. 2A). The related succinimide-functionalized derivatives **9–10** and **13–14** showed no changes over a period of 24 hours. Thus, the hydrolysis can be clearly attributed to the maleimide moiety.

Next, the binding of the maleimide-functionalized complexes to the thiol group of human serum albumin (HSA) was investigated. Therefore, compounds **7–8** and **11–12** were incubated with HSA and the decrease of the peak area of the unbound drug was monitored (Fig. 2B, Table S19†).

In general, the cisplatin-based compounds **7–8** reacted faster with HSA than the oxaliplatin-derivatives **11–12**. Concerning the methoxido- and acetato-functionalized compounds, no distinct trend regarding albumin binding could be observed. An increase in HSA equivalents also accelerated the binding as expected. In comparison to the previously published data on bis-functionalized maleimide compounds,^26^ the half-life time of the mono-functional complexes is approximately doubled. This is in line with the lower number of maleimide groups able to bind to albumin.

### Binding studies in serum

**Fig. 3:**
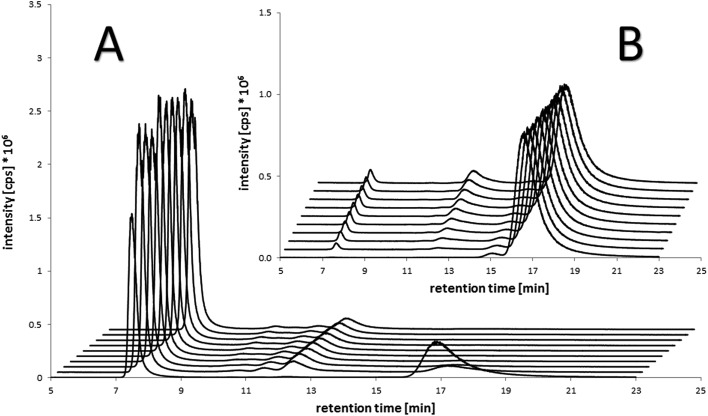
Platinum-SEC-ICP-MS chromatograms of the incubation of (A) the maleimide-containing oxaliplatin(iv) compound **12** (100 μM) in fetal calf serum after 10 min (chromatogram in front) and each 140 min up to 21 h in comparison to (B) the succinimide-containing reference compound **13** under the same conditions. Albumin retention: 7.6 min (size ladder see Table S11†).

To ascertain the actual binding target, the binding rate and the stability of the formed species under biological conditions, the compounds were incubated with fetal calf serum at 37 °C and sulfur- and platinum-containing (macro)molecules were measured with size exclusion chromatography inductively coupled plasma mass spectrometry (SEC-ICP-MS) over 21 h. Already at the first scan (∼5–10 min after incubation) ∼50% of the maleimide-containing compounds **7–8** and **11–12** were bound to the albumin fraction at 7.6 min (see Table S11† for the corresponding size ladder). At the next time point after 140 min, in all cases, >80% of the drug was bound to albumin. In addition, a small peak with <10% of platinum was observed in these incubation experiments in the low-molecular range (∼12 min retention time), presumably due to adducts on low-molecular weight thiol-containing molecules *e.g.* glutathione and cysteine (Fig. 3). Thus, the fast as well as specific binding to serum albumin was verified. These results are also in good accordance to the distribution of reduced thiol groups in human plasma with more than >400 μM originating from albumin and only <20 μM in the case of low-molecular-weight thiol-containing molecules due to its presence mainly in its oxidized form.^32^ Moreover, these analyses indicated very stable albumin adducts and no release of low-molecular-weight platinum compounds due to reduction or instability, as no significant changes in the signals between 4 and 21 h occurred. In contrast, the succinimide-functionalized complexes **9–10** and **13–14** showed negligible protein binding ability during 21 h (Fig. 3B), proving the very high specificity of the maleimide moiety towards albumin in serum. Finally, dynamic light scattering (DLS) measurements of compounds **8** and **12** incubated with albumin were performed, which indicated that the binding of the drugs did not impact on the size of the protein (see Fig. S3†).

### Anticancer activity *in vivo*

**Fig. 4:**
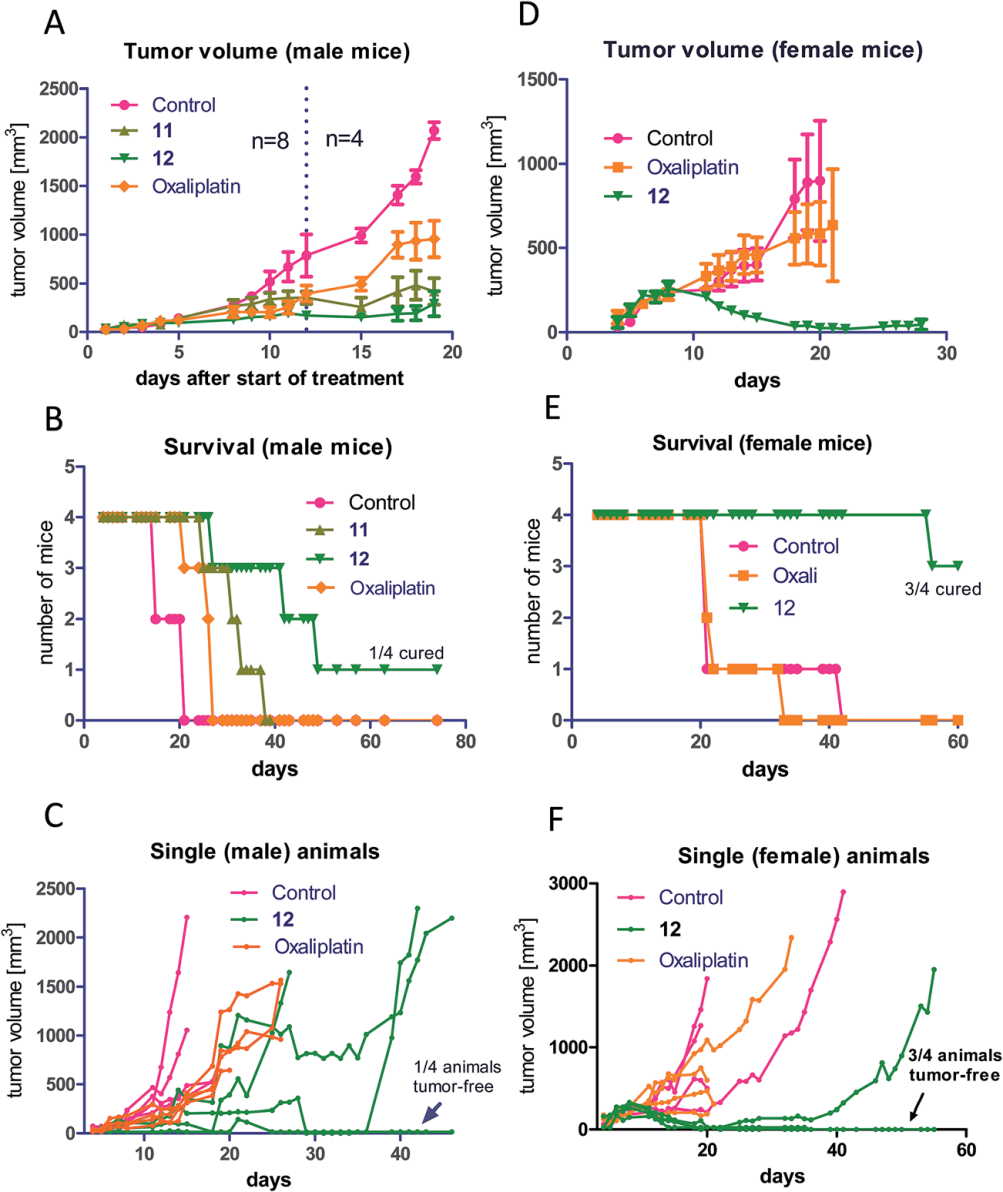
*In vivo* anticancer activity of **11** and **12** in comparison to oxaliplatin: CT-26 cells were injected subcutaneously into the right flank of male BALB/c mice. Mice were treated twice a week (Monday and Thursday) i.v. with concentrations equimolar to 9 mg kg^–1^ oxaliplatin (each experimental group contained four animals). Tumor volumes were calculated as described in the experimental part. The experiment was performed twice with two different endpoints: overall survival *vs.* tissue collection 12 days after the first treatment. (A) shows the data of both experiments pooled together. The dashed line indicates the date of section of 4 mice of each treatment group. (B) shows the individual survival of the other 4 mice of each group, while (C) depicts the individual tumor growth of these animals. (D) shows the tumor volume, (E) the overall survival and (F) the individual survival gained from groups of four female mice, which were treated with the same scheme as the male animals. Data are means ± SEM.

As a next step, the anticancer activity of **8**, **11** and **12** in comparison to cis- and oxaliplatin was tested against subcutaneous CT-26 tumors in male BALB/c mice (due to the albumin binding, the new compounds were widely inactive in cell culture; data not shown). Unexpectedly, despite their similar behavior in the binding studies, distinct differences with regard to onset and strength of anticancer activity between the different maleimide compounds were observed: as such, only the two oxaliplatin derivatives proved to be superior to their respective platinum(ii) compound (Fig. 4A), while no difference in the case of the cisplatin derivative **8** was observed (Fig. S4†). Moreover, the oxaliplatin acetato derivative **12** was distinctly more effective than the methoxido derivative **11** at equimolar concentrations probably due to a distinctly earlier onset of activity (day 5 in the case of **12** compared to day 8 in the case of **11** and oxaliplatin). This resulted not only in prolonged overall survival but also cured one of the treated animals (Fig. 4B). Fig. 4C, where the individual tumor responses are depicted, shows that this effectiveness is based on both long time frames of disease stabilization and tumor shrinkage. Noteworthy, compound **12** was found to be even more effective in female animals, where treatment cured 3 out of 4 animals (Fig. 4D–F). In contrast, oxaliplatin was widely ineffective at the given concentration, which was probably based on the occurrence of very strong adverse effects, which also made the early sacrifice due to the bad physical condition of the treated mice necessary. Enhanced sensitivity of females to oxaliplatin has been also reported for patients,^33–35^ indicating gender differences for this drug due to yet unexplored reasons.

**Fig. 5:**
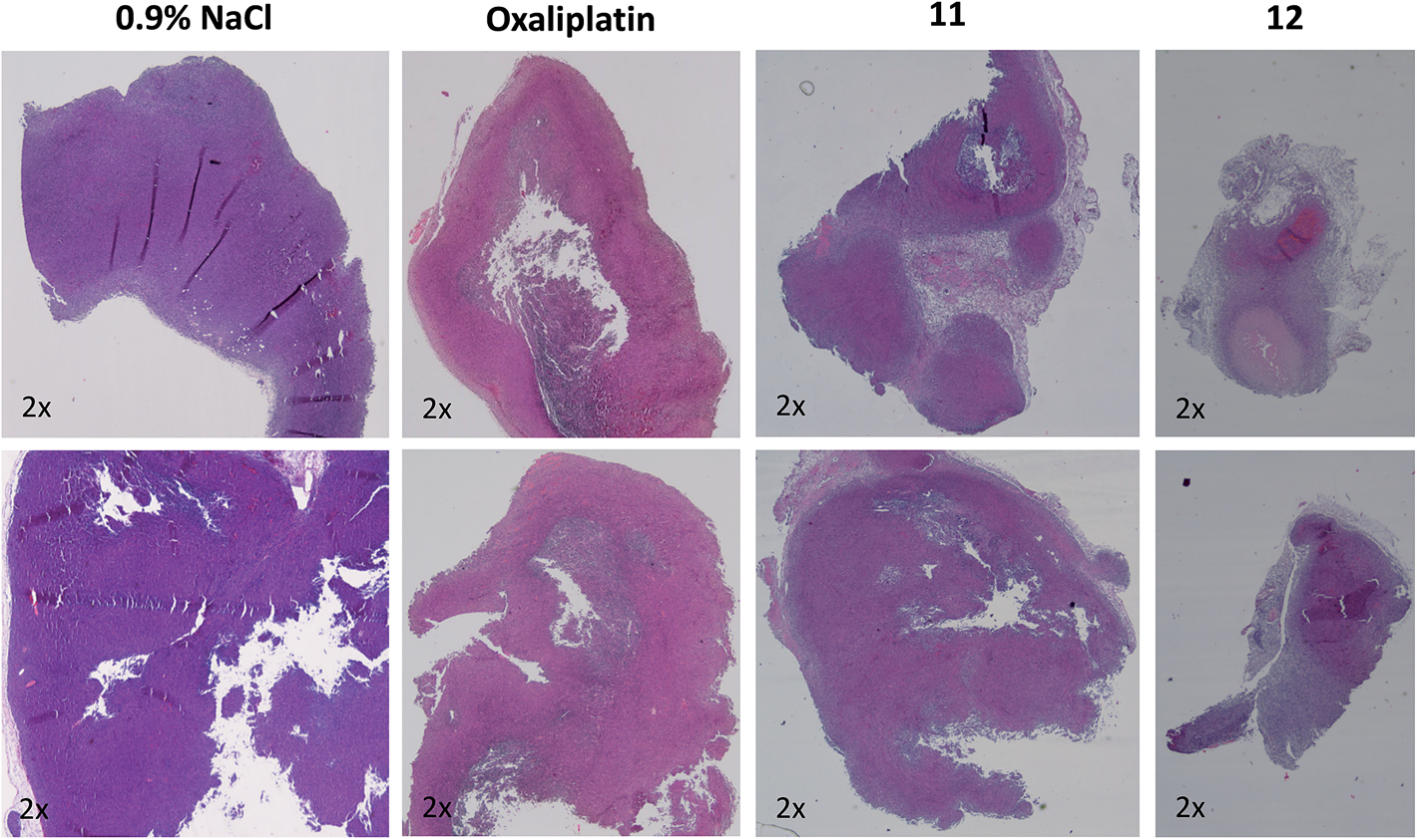
Histological evaluation of tumors treated with **11** and **12** in comparison to oxaliplatin. Tumors were harvested on day 12 of the male BALB/c experiment shown in Fig. 4A. After formalin fixation and paraffin embedding, tissue slices were prepared and H/E-stained. Pictures were taken using a transmitted light microscope suitable for histological imaging, at the smallest magnification (2×), using the same settings for every slice. Two representative tumors of every treatment group are shown.

In order to better understand the observed differences in the activity of our compound panel, in a second experiment male CT-26-bearing animals were sacrificed on day 12 (24 h after the last treatment) and paraffin-embedded tumors were evaluated by H/E stain. In the case of **12**, the treatment already resulted (comparable to the first experiment) in complete remission of one animal, allowing the histological analysis of 3 animals only. In addition, as depicted in Fig. 5, the tumors of mice treated with **12** were clearly smaller than the ones of the other treatment groups. Microscopy analyses of the tissue morphology (by counting) revealed that all drugs of our test panel induced a significant increase of the apoptotic cell fraction (by one-way ANOVA and Dunnett posttest; Fig. S5†). With the exception of **12**, the strength of apoptosis induction was also in good agreement with the anticancer activity observed at day 12 (see Fig. 4A): thus, highest (similar) apoptosis levels were observed for the oxaliplatin methoxido compound **11** and oxaliplatin, while the levels for the cisplatin acetato compound **8** were about 30% lower than for cisplatin. Astonishingly, the most active oxaliplatin acetato compound **12** with distinctly smaller tumors at day 12 (Fig. 5), had lower levels of apoptotic cells compared to the methoxido derivative **11**. One possible explanation is the earlier onset of activity of **12** (which is also in line with the very small sizes of the collected tumors), indicating that the peak of anticancer activity had been reached earlier and the activity was already declining at the time point of tumor collection. Furthermore, the histological evaluation of tumor material was only possible for 3 animals because the fourth mouse already experienced a complete remission at time of section (which means that the best responding animal was not available for evaluation). Noteworthy, in the three remaining tumors treated with **12** a visible reduction in the mitotic cell fraction was still found (Fig. S5†). However, due to the very low percentage of mitotic cells, this effect did not reach statistical significance.

### Platinum tissue distribution after short-term treatment

**Fig. 6:**
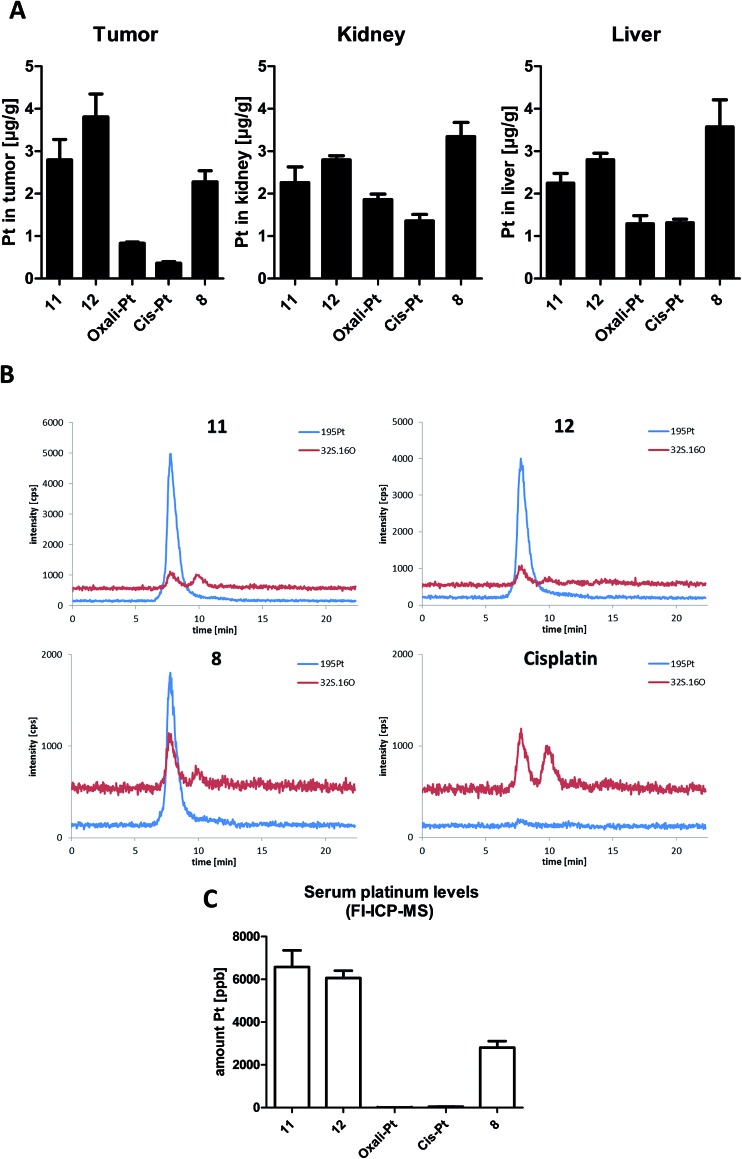
*In vivo* platinum distribution of **8**, **11**, **12**, cis- and oxaliplatin 24 h after single treatment: CT-26 cells were injected subcutaneously into the right flank of male BALB/c mice. Mice were treated once i.v. with 9 mg kg^–1^ oxaliplatin and the equimolar concentrations in the case of **11** and **12** as well as 3 mg kg^–1^ cisplatin and an equimolar dose in the case of **8**. After 24 h, animals were anesthetized, sacrificed by heart puncture and tumor, liver, kidney tissue as well as serum were collected. Each experimental group contained four animals. (A) Organ platinum-distribution measured by ICP-MS. Data are expressed as mean ± standard deviation. (B) Platinum-SEC-ICP-MS measurements of serum samples (C) serum platinum levels measured by FI-ICP-MS. Data are expressed as mean ± standard deviation.

It is well accepted that albumin targeting distinctly impacts on the plasma half-life time as well as tissue distribution.^36^ Especially, the enhanced accumulation due to the EPR effect makes this form of tumor-targeting very interesting for the development of novel cancer therapeutics.^13^ As our novel compounds have a double prodrug nature (albumin-binding and the need of activation by reduction), we were interested in the drug tissue distribution after treatment. To this end, male CT-26-bearing BALB/c mice were treated once with our compounds followed by sample collection 24 h later. Fig. 6A shows the comparison of platinum levels in tumor, kidney and liver tissues of the treated animals. In accordance to many other non-targeted drugs,^37–40^ for both platinum(ii) drugs the intratumoral drug levels were distinctly lower than in the liver and kidney (which usually have very high drug levels due to their important function in metabolism and excretion). Interestingly, although more than 2-fold higher platinum levels were applied in the case of oxaliplatin and its derivatives (**11** and **12**) compared to cisplatin and its prodrug **8**, the tested organ levels were in a similar range. This indicates that there are *per se* strong differences in the pharmacological behavior of the oxali- and cisplatin. With regard to the impact of the maleimide moiety, all three platinum(iv) prodrugs showed distinctly enhanced tumor levels in comparison to the respective platinum(ii) compound. However, only in the case of **12**, tumor levels significantly exceeded the ones of the kidney and liver (*p* > 0.05 by one-way-ANOVA), while they were not significantly different or even lower in the case of **11** and **8**, respectively. Noteworthy, despite the lower applied dose (equimolar to 3 mg kg^–1^ cisplatin) in the case of the cisplatin derivative **8**, the animals in this treatment group were characterized by organ platinum levels in the range of the oxaliplatin derivatives and even higher platinum content in liver and kidney tissue. This is remarkable as parallel SEC-ICP-MS and flow-injection-(FI)-ICP-MS investigations of the serum samples 24 h after treatment revealed that, in accordance to the applied doses, the total Pt levels of the oxaliplatin derivatives **11** and **12** were more than 2-fold higher than the one of the cisplatin derivative **8** (Fig. 6B and C). Notably, at this time point the serum concentrations of both platinum(ii) drugs were already very low.

In general, the higher tissue and serum levels of the maleimide compounds were not unexpected, as albumin binding of drugs (Fig. 6B) is known to hamper renal excretion^12^ and, by this, results in a prolonged plasma half-life time. However, our data indicate that **12** is superior to **11** and especially **8** with respect to tumor-targeting indicating distinct differences in the pharmacological behavior and susceptibility to the EPR effect.

### Reduction experiments

**Fig. 7:**
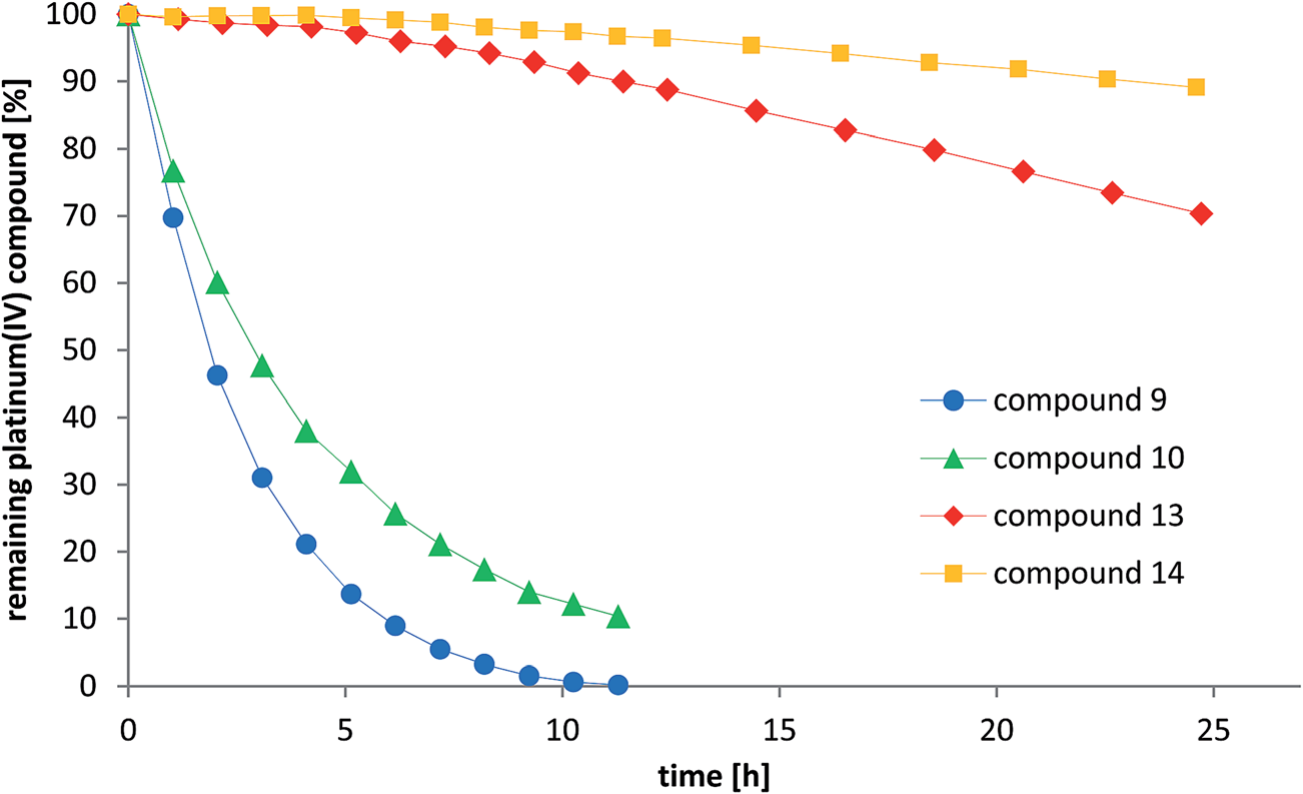
Reduction of the compounds **9–10** and **13–14** with 10 eq. ascorbic acid in phosphate buffer at pH = 7.4 monitored by RP-HPLC.

As one explanation of the observed differences between the compounds might be altered reducibility, we investigated the reduction rate in the presence of ascorbic acid in phosphate-buffered solution at pH = 7.4 using NMR spectroscopy and HPLC measurements. To reduce the number of additional signals from hydrolyzed maleimide species in the spectra, only the succinimide-functionalized compounds **9–10** and **13–14** were evaluated in these experiments (Fig. 7 and S6†). For both NMR and HPLC measurements, the same complex concentrations (1 mM) and 10 equivalents ascorbic acid were used. The data showed that the cisplatin-derived compounds (**9–10**) are reduced much faster than the oxaliplatin-based complexes (**13–14**), which can be explained by the faster electron transfer *via* the chloride ligands.^41^ Additionally, platinum(iv) compounds bearing an acetato ligand (**10**, **14**) were more stable than those with a methoxido ligand (**9**, **13**). Comparison of the HPLC data and the NMR measurements showed very similar tendencies. Overall, there is a strong correlation between the reduction data and the *in vivo* activity. As such, the fast reduction of the cisplatin derivatives correlates with the low *in vivo* activity of **8** and relatively low plasma levels after 24 h. In contrast, the oxaliplatin derivatives were characterized by very high inertness towards reduction (even more pronounced for the acetato derivative **12**) together with higher plasma levels after 24 h and strongly enhanced anticancer activity.

To further investigate the reduction process, the release of the HSA-bound platinum was investigated. To this end, the cisplatin-based maleimide compound **8** was incubated with 5 eq. of HSA to achieve maximal binding and remaining low-molecular-weight platinum was removed by filtration through Sephadex. Subsequently, the obtained HSA-platinum fraction was incubated with 10 eq. of ascorbic acid for 24 h for reduction. After removal of the high-molecular-weight fraction, the solution was analyzed by FI-ICP-MS and RP-HPLC coupled to an ICP-MS instrument. The data clearly revealed the formation of a platinum(ii) reduction product, which elutes at the same time as the cisplatin reference compound (see Fig. S7†). Notably, the detected amount of the new platinum(ii) species was only ∼10% of the total platinum content, which possibly can be explained by non-covalent binding of the released cisplatin to albumin.^42,43^

## Conclusions

Besides the rapid development of drug resistance, adverse effects are still the major limitation for successful chemotherapy in the advanced stage. In the case of platinum drugs especially nephrotoxicity, neurotoxicity, ototoxicity, and myelosuppression are most problematic.^9,44^ In order to address these issues, more kinetically inert platinum(iv) drugs were developed with satraplatin as the furthest clinically developed representative.^45^ However, even this promising compound, while being less toxic, failed in a clinical phase III trial, because it did not reach the endpoint of improved overall survival.^46^ This might also be associated with the fact that despite being a prodrug, satraplatin has no tumor-targeting properties. In addition, it is still questionable, whether the proposed activation of the drug by reduction to the active platinum(ii) complex is a solely tumor-specific process, as there are several indications for reduction of satraplatin *e.g.* in the red blood cell compartments by reaction with hemoglobin^10^ or in the liver by metabolizing enzymes.^11^ Consequently, in the last few years we focused on the development of novel tumor-targeted platinum(iv) drugs.^26^ In this study, we present the first mono-functionalized maleimide-containing platinum(iv) complexes. The panel of newly synthesized complexes showed that there are distinct differences, *e.g.* in the *in vivo* anticancer activity, not only between cis- and oxaliplatin derivatives, but also between closely related compounds, which only differ in one axial ligand. Subsequent analyses revealed that this is most likely not based on the aqueous stability or albumin binding velocity of the compounds. However, strong differences in the reducibility of the platinum(iv) cores were observed, which could provide at least some explanation for the observed effects *in vivo*. Thus, the cisplatin derivatives were characterized by much faster reduction compared to the oxaliplatin analogues. This is in line with recent literature reports, which show that complexes containing equatorial chlorido ligands have distinctly shorter half-lives in the presence of ascorbate in aqueous solution compared to their oxalato or cyclobutane dicarboxylic acid analogues.^47,48^ Noteworthy, the exchange of the methoxido to an acetato ligand further increased the inertness towards reduction. In addition to the reducibility, also the tumor-targeting potential of the albumin-bound drugs 24 h after i.v. administration distinctly varied. This is unexpected as the detected serum platinum levels were in line with the applied drug dose and SEC-ICP-MS serum incubation experiments revealed that the stability of the albumin adducts did not differ between cis- and oxaliplatin derivatives. This indicates that the observed differences in tumor-targeting are not based on interaction with reductants in the serum but on other factors. Hence, for example, the different reducibility of the drugs might impact on their metabolization in the body, which would also fit to the comparably high platinum content of **8** observed in the main organs responsible for drug elimination, namely liver and kidney.

In general, the differences in the *in vivo* anticancer activity between the two oxaliplatin derivatives **11** and **12** are remarkable. Although they behaved widely similar with respect to their chemical properties under cell-free conditions (drug stability, albumin binding, reducibility *etc.*), compound **12** resulted in the complete and long lasting response of ∼25% of male and ∼75% female animals (the animals are still tumor-free more than 1 year after therapy). In contrast, **11** just led to disease stabilization but not to tumor regression. This is also surprising as both derivatives were present at similar serum levels after 24 h. However, some differences were observed with respect to the tissue levels of the drugs, where **12** was superior to **11**. This might indicate that the drugs differ in their extravasation potency *e.g.* by different EPR efficacy of the drug–albumin-adducts or by different affinity for glycoprotein 18 and 30 (gp18 and gp30), two membrane-associated proteins, which are involved in the recognition of conformationally modified albumin molecules.^13,18^ As both fields – albumin metabolism and *in vivo* activation by reduction of platinum(iv) drugs – are surprisingly unexplored, more in-depth studies are necessary to fully understand the impact of albumin-targeting on the biology of anticancer agents.

Taken together, the here presented study reports the first mono-maleimide platinum(iv) prodrugs of cis- and oxaliplatin and revealed the distinct impact of small structural modifications on their *in vivo* biology and anticancer activity. By this approach, we were able to identify a novel, very potent lead compound, which exerts anticancer activity superior to oxaliplatin by inducing several complete responses (especially in females). Therefore, this novel drug has now been selected for further (pre)clinical development towards clinical phase I trials.
