# Supplementary material for: An albumin-based tumor-targeted oxaliplatin prodrug with distinctly improved anticancer activity in vivo
Source: Chem Sci. 2016 Dec 15;8(3):2241–50. doi: 10.1039/c6sc03862j (PMC5409245; doi:10.1039/c6sc03862j)
Supplement: Supplementary file 1 [file SC-008-C6SC03862J-s001.pdf]

Electronic Supplementary Material (ESI) for Chemical Science.
This journal is © The Royal Society of Chemistry 2016

Electronic Supporting Information (ESI)

An albumin-based tumor-targeted oxaliplatin prodrug with distinctly improved anticancer activity *in vivo*

Josef Mayr[a], Petra Heffeter*[bc], Diana Groza[b], Luis Galvez[d], Gunda Koellensperger[d], Alexander Roller[a], Beatrix Alte[ab], Melanie Haider[b], Walter Berger[bc], Christian R. Kowol*[ac], Bernhard K. Keppler[ac]

[a] University of Vienna, Institute of Inorganic Chemistry, Waehringer Strasse 42, A-1090, Vienna, Austria.

[b] Institute of Cancer Research and Comprehensive Cancer Center, Medical University of Vienna, Borschkegasse 8a, A-1090, Vienna, Austria.

[c] Research Platform ''Translational Cancer Therapy Research'' University of Vienna, Waehringer Strasse 42, A-1090, Vienna, Austria.

[d] University of Vienna, Institute of Analytical Chemistry, Waehringer Strasse 38, A-1090, Vienna, Austria.

Table of Content

Materials and methods 4

Synthesis 5

General procedure for the synthesis of compounds **7–14**: 5

Purification of compounds **7–14** by preparative RP-HPLC: 5

Characterization 6

Compound **7** 6

Compound **8** 6

Compound **9** 7

Compound **10** 7

Compound **11** 8

Compound **12** 8

Compound **13** 9

Compound **14** 9

Single crystal X-ray crystallography 11

RP-HPLC stability and binding studies 17

SEC-ICP-MS studies 17

SEC-ICP-MS stability and FCS binding studies 17

SEC-ICP-MS measurements of *in vivo* serum samples 19

DLS measurements 21

***In vivo* studies** 22

Cell culture 22

Animals 22

CT-26 experiments 22

Organ distribution experiments 23

Statistics 23

Determination of the platinum concentration in mice tissues by ICP-MS 23

Reduction experiments 24

Platinum release experiment 25

Tables and figures 27

References 30

Materials and methods

Potassium tetrachloridoplatinate (K_2PtCl_4) was purchased from Johnson Matthey (Switzerland). Water for synthesis was taken from a reverse osmosis system and distilled twice before use. For HPLC measurements Milli-Q water (18.2 MΩ·cm, Merck Milli-Q Advantage, Darmstadt, Germany) was used. Other chemicals and solvents were purchased from commercial suppliers (Sigma Aldrich, Merck, Acros, Fluka and Fisher Scientific). Cisplatin[1] (**1**) and oxaliplatin[2] (**2**) were prepared according to literature procedures. Thereafter, the complexes were oxidized with hydrogen peroxide (50%) using either methanol[3] or acetic acid[4] as a solvent to yield the unsymmetrically oxidized platinum(IV) precursors **3**–**6**. The maleimide- and succinimide-functionalized ligands were prepared as recently published.[5] Electrospray ionization (ESI) mass spectra were recorded on a Bruker amaZon SL ion trap mass spectrometer in positive and/or negative mode by direct infusion. High resolution mass spectra were measured on a Bruker maXis™ UHR ESI time of flight mass spectrometer. All mass spectra were recorded at the Mass Spectrometry Centre of the University of Vienna. One- and two-dimensional ^{1}H-, ^{13}C-, ^{15}N- and ^{195}Pt- NMR spectra were recorded on a Bruker Avance III 500 MHz spectrometer at 500.10 (^{1}H), 127.75 (^{13}C), 50.68 (^{15}N), and 107.51 (^{195}Pt) MHz at 298 K. For ^{1}H- and ^{13}C-NMR spectra the solvent residual peak was taken as internal reference, whereas ^{195}Pt-shifts were referenced relative to external K_2PtCl_4 and ^{15}N-shifts relative to external NH_4Cl. Elemental analysis measurements were performed on a Perkin Elmer 2400 CHN Elemental Analyzer at the Microanalytical Laboratory of the University of Vienna.

Synthesis

General procedure for the synthesis of compounds 7–14:

The unsymmetrically, oxidized platinum(IV) compound (**3**–**6**) and the corresponding isocyanate were transferred into a Schlenk flask and set under argon atmosphere. After the addition of dry DMF, the suspension was stirred overnight. A remaining precipitate was filtered, before the DMF was removed under reduced pressure. The residue were taken up as a suspension in methanol and fully precipitated by addition of diethylether. After a few hours in the fridge the crude product was filtered off, washed with diethylether and dried under reduced pressure. The solid was taken up in water and filtered before it was purified by preparative RP-HPLC. Collected product fractions were concentrated, to remove methanol, thereafter lyophilized and dried under reduced pressure.

Purification of compounds 7–14 by preparative RP-HPLC:

All compounds were purified by preparative RP-HPLC using a Waters XBridge C18 column on an Agilent 1200 Series system. Milli-Q water, containing 0.1% formic acid, and methanol were used as eluents.

Characterization

Compound 7

(*OC*-6-44)-Diamminedichloridomethoxido[2-(2,5-dioxo-2,5-dihydro-1*H*-pyrrol-1-yl)ethylcarbamato]platinum(IV)

The compound was synthesized from (*OC*-6-44)-diamminedichloridohydroxidomethoxidoplatinum(IV) (140 mg, 0.40 mmol) and 1-(2-isocyanatoethyl)-1*H*-pyrrole-2,5-dione (2 eq., 134 mg, 0.80 mmol) in 3 mL DMF (abs.). Purification via preparative RP-HPLC, isocratic with 8% methanol, yielded a pale yellow solid; Yield: 70.5 mg (18%); ^{1}H-NMR (DMSO-d_6): δ = 6.98 (s, 2H, C*H*), 6.38 (s, 1H, N*H*), 6.17–5.73 (m, 6H, NH_3), 3.46–3.36 (m, 2H, NCH_2), 3.06–2.96 (m, 2H, NHCH_2), 2.63 (bs, 3H, CH_3) ppm; ^{13}C-NMR (DMSO-d_6): δ = 171.1 (*C*OCH), 164.8 (*C*ONH), 134.5 (*C*H), 60.5 (CH_3), 39.2 (NHCH_2), 37.5 (NCH_2) ppm; ^{15}N{^{1}H}-NMR (DMSO-d_6): δ = 59.5 (*N*H), -31.7 (NH_3) ppm; ^{195}Pt-NMR (DMSO-d_6): δ = 2642 (major), 2629 (minor) ppm; Two separated platinum signals were observed due to conformational isomers of the carbamate group.[6] HRMS (ESI-TOF): calcd. for [$C_8H_{16}Cl_2N_4O_5Pt$-Na^+]$^+$: 536.0043, found: 536.0028; elemental analysis calcd. for $C_8H_{16}Cl_2N_4O_5Pt{\cdot}0.5H_2O$: C: 18.36, H: 3.27, N: 10.71, found: C: 18.38, H: 2.92, N: 10.32

Compound 8

(*OC*-6-44)-Acetatodiamminedichlorido[2-(2,5-dioxo-2,5-dihydro-1*H*-pyrrol-1-yl)ethylcarbamato]platinum(IV)

The compound was synthesized from (*OC*-6-44)-acetatodiamminedichloridohydroxidoplatinum(IV) (450 mg, 1.20 mmol) and (2 eq., 398 mg, 2.39 mmol) in 5 mL DMF (abs.). Purification via preparative RP-HPLC, isocratic with 3% methanol, yielded a pale yellow solid; Yield: 267.0 mg (42%); ^{1}H-NMR (DMSO-d_6): δ = 6.98 (s, 2H, C*H*), 6.80–6.37 (m, 6H, NH_3), 6.67 (bs, 1H, N*H*), 3.45–3.37 (m, 2H, NCH_2), 3.07–2.99 (m, 2H, NHCH_2), 1.90 (s, 3H, CH_3) ppm; ^{13}C-NMR (DMSO-d_6): δ = 178.2 (*C*OCH_3), 171.1 (*C*OCH), 163.8 (*C*ONH), 134.5 (*C*H), 38.9 (NHCH_2), 37.4 (NCH_2), 22.8 (CH_3) ppm; ^{15}N{^{1}H}-NMR (DMSO-d_6): δ = 59.2 (*N*H), -39.4 (NH_3) ppm; ^{195}Pt-NMR (DMSO-d_6): δ = 2880 (major), 2860 (minor) ppm; HRMS (ESI-TOF): calcd. for [$C_9H_{16}Cl_2N_4O_6Pt$-Na^+]$^+$: 564.9944, found: 564.9977;

elemental analysis calcd. for $C_9H_{16}Cl_2N_4O_6Pt{\cdot}1.5H_2O$: C: 18.99, H: 3.36, N: 9.84, found: C: 18.73, H: 3.18, N: 9.70

Compound 9

(*OC*-6-44)-Diamminedichloridomethoxido[2-(2,5-dioxopyrrolidin-1-yl)ethyl)carbamato]platinum(IV)

The compound was synthesized from (*OC*-6-44)-diamminedichloridohydroxidomethoxidoplatinum(IV) (200 mg, 0.57 mmol) and 1-(2-isocyanatoethyl)pyrrolidine-2,5-dione (1.5 eq., 145 mg, 0.86 mmol) in 3 mL DMF (abs.). Purification via preparative RP-HPLC, isocratic with 5% methanol, yielded a pale yellow solid; Yield: 124.2 mg (42%); ^{1}H-NMR (DMSO-d_6): δ = 6.32 (s, 1H, N*H*), 6.20–5.70 (m, 6H, NH_3), 3.47–3.29 (m, 2H, NCH_2), 3.10–2.92 (m, 2H, NHCH_2), 2.63 (bs, 3H, CH_3), 2.58 (bs, 4H, COCH_2) ppm; ^{13}C-NMR (DMSO-d_6): δ = 177.9 (*C*OCH$_2$), 164.9 (*C*ONH), 60.5 (*C*H$_3$), 38.3 (bs, NH*C*H$_2$, N*C*H$_2$), 28.1 (CO*C*H2) ppm; ^{195}Pt-NMR (DMSO-d_6): δ = 2642 (major), 2629 (minor) ppm; HRMS (ESI-TOF): calcd. for $[C_8H_{18}Cl_2N_4O_5Pt\text{-}Na^+]^+$: 538.0200, found: 538.0195; elemental analysis calcd. for $C_8H_{18}Cl_2N_4O_5Pt$: C: 18.61, H: 3.51, N: 10.85, found: C: 18.73, H: 3.66, N: 10.22

Compound 10

(*OC*-6-44)-Acetatodiamminedichlorido[2-(2,5-dioxopyrrolidin-1-yl)ethyl)carbamato]platinum(IV)

The compound was synthesized from (*OC*-6-44)-acetatodiamminedichloridohydroxidoplatinum(IV) (600 mg, 1.60 mmol) and (1.5 eq., 402 mg, 2.39 mmol) in 5 mL DMF (abs.). Purification via preparative RP-HPLC, with a gradient from 5–10.5% methanol over 10 min, yielded a pale yellow solid; Yield: 232.2 mg (27%); ^{1}H-NMR (DMSO-d_6): δ = 6.89–6.33 (m, 7H, NH_3, N*H*), 3.41–3.34 (m, 2H, NCH_2), 3.03 (q, *J* = 6.2 Hz, 2H, NHCH_2), 2.59 (bs, 4H, COCH_2), 1.90 (s, 3H, CH_3) ppm; ^{13}C-NMR (DMSO-d_6): δ = 178.2 (*C*OCH$_3$), 177.8 (*C*OCH$_2$), 163.9 (*C*ONH), 38.2 (NH*C*H$_2$, N*C*H$_2$), 28.1 (CO*C*H$_2$), 22.8 (*C*H$_3$) ppm; ^{195}Pt-NMR (DMSO-d_6): δ = 2880 (major), 2859 (minor) ppm; HRMS (ESI-TOF): calcd. for $[C_9H_{18}Cl_2N_4O_6Pt\text{-}Na^+]^+$: 567.0100, found: 567.0138; elemental analysis calcd. for $C_9H_{18}Cl_2N_4O_6Pt{\cdot}H_2O$: C: 19.23, H: 3.59, N: 9.96, found: C: 19.17, H: 3.35, N: 9.88

Compound 11

(*OC*-6-34)-[(1*R*,2*R*)-Cyclohexane-1,2-diamine]methoxidooxalato[2-(2,5-dioxo-2,5-dihydro-1*H*-pyrrol-1-yl)ethylcarbamato]platinum(IV)

The compound was synthesized from (*OC*-6-44)-[(1*R*,2*R*)-cyclohexane-1,2-diamine]hydroxidomethoxidooxalatoplatinum(IV) (170 mg, 0.38 mmol) and 1-(2-isocyanatoethyl)-1*H*-pyrrole-2,5-dione (2 eq., 127 mg, 0.76 mmol) in 7 mL DMF (abs.). Purification via preparative RP-HPLC, with a gradient from 15–35% methanol in 10 min, yielded a white solid; Yield: 88.7 mg (38%); ^{1}H-NMR (DMSO-d_6): δ = 9.85–9.55 (m, 1H, NH_2), 8.33–8.09 (m, 1H, NH_2), 7.79–7.59 (m, 1H, NH_2), 7.07–6.83 (m, 1H, NH_2), 6.98 (s, 2H, COC*H*), 6.44 (s, 1H, N*H*), 3.55–3.45 (m, 1H, NCH_2), 3.39–3.29 (m, 1H, NCH_2), 3.15–3.05 (m, 1H, NHCH_2), 3.00–2.90 (m, 1H, NHCH_2), 2.63–2.43 (m, 2H, CH_{dach}), 2.41 (s, 3H, CH_3), 2.19–2.07 (m, 1H, C$H_{2,\text{dach}}$), 2.07–1.96 (m, 1H, C$H_{2,\text{dach}}$), 1.60–1.42 (m, 3H, C$H_{2,\text{dach}}$), 1.39–1.27 (m, 1H, C$H_{2,\text{dach}}$), 1.25–1.07 (m, 2H, C$H_{2,\text{dach}}$) ppm; ^{13}C-NMR (DMSO-d_6): δ = 171.0 (*C*OCH), 165.2 (*C*ONH), 163.7 (*C*O_{ox}), 163.6 (*C*O_{ox}), 134.4 (CO*C*H), 61.3 (*C*H_{dach}), 59.5 (*C*H_{dach}), 57.4 (*C*H_3), 39.0 (NH*C*H_2), 37.4 (N*C*H_2), 30.6 (*C*$H_{2,dach}$), 23.7 (*C*$H_{2,\text{dach}}$), 23.6 (*C*$H_{2,\text{dach}}$) ppm; ^{15}N{^{1}H}-NMR (DMSO-d_6): δ = 60.9 (*N*H), -6.4 (*N*H_2), -1.5 (*N*H_2) ppm; ^{195}Pt-NMR (DMSO-d_6): δ = 3001 (major), 2995 (minor) ppm; HRMS (ESI-TOF): calcd. for $[C_{16}H_{24}N_4O_9Pt\text{-}Na^+]^+$: 634.1089, found: 634.1089; elemental analysis calcd. for $C_{16}H_{24}N_4O_9Pt{\cdot}H_2O$: C: 30.53, H: 4.16, N: 8.90, found: C: 30.29, H: 4.08, N: 8.75

Compound 12

(*OC*-6-34)-Acetato[(1*R*,2*R*)-cyclohexane-1,2-diamine]oxalato[2-(2,5-dioxo-2,5-dihydro-1*H*-pyrrol-1-yl)ethylcarbamato]platinum(IV)

The compound was synthesized from (*OC*-6-44)-acetato[(1*R*,2*R*)-cyclohexane-1,2-diamine]hydroxidooxalatoplatinum(IV) (200 mg, 0.42 mmol) and 1-(2-isocyanatoethyl)-1*H*-pyrrole-2,5-dione (2 eq., 140 mg, 0.85 mmol) in 2 mL DMF (abs.). Purification via preparative RP-HPLC, isocratic with 15% methanol, yielded a white solid; Yield: 111.2 mg (41%); ^{1}H-NMR (DMSO-d_6): δ = 9.63 (bs, 1H, NH_2), 8.74–8.00 (m, 3H, NH_2), 6.99 (s, 2H, COC*H*), 6.79 (t, *J* = 5.9 Hz, 1H, N*H*), 3.58–3.45 (m, 1H, NCH_2), 3.42–3.29 (m, 1H, NCH_2), 3.19–3.08 (m, 1H, NHCH_2), 3.01–2.89 (m, 1H, NHCH_2), 2.70–2.60 (m, 1H, CH_{dach}), 2.60–2.50 (m, 1H, CH_{dach}), 2.15

(bs, 2H, C$H_{2,dach}$), 1.95 (s, 3H, CH_3), 1.59–1.10 (m, 6H, C$H_{2,dach}$) ppm; ^{13}C-NMR (DMSO-d_6): δ = 178.4 (COCH_3), 171.0 (COCH), 164.2 (CONH), 163.3 (CO_{ox}), 134.5 (COCH), 61.1 (CH_{dach}), 60.7 (CH_{dach}), 39.0 (NHCH_2), 37.2 (NCH_2), 31.0 ($CH_{2,dach}$), 30.9 ($CH_{2,dach}$), 23.5 ($CH_{2,dach}$), 23.4 ($CH_{2,dach}$), 22.9 (CH_3) ppm; ^{195}Pt-NMR (DMSO-d_6): δ = 3236 (major), 3227 (minor) ppm; HRMS (ESI-TOF): calcd. for $[C_{17}H_{24}N_4O_{10}Pt\text{-}Na^+]^+$: 662.1038, found: 662.1033; elemental analysis calcd. for $C_{17}H_{24}N_4O_{10}Pt{\cdot}H_2O$: C: 31.05, H: 3.99, N: 8.52, found: C: 30.96, H: 3.91, N: 8.44

Compound 13

(*OC*-6-34)-[(1*R*,2*R*)-Cyclohexane-1,2-diamine]methoxidooxalato[2-(2,5-dioxopyrrolidin-1-yl)ethyl)carbamato]platinum(IV)

The compound was synthesized from (*OC*-6-44)-[(1*R*,2*R*)-cyclohexane-1,2-diamine]hydroxidomethoxidooxalatoplatinum(IV) (200 mg, 0.45 mmol) and 1-(2-isocyanatoethyl)pyrrolidine-2,5-dione (2 eq., 151 mg, 0.90 mmol) in 3 mL DMF (abs.). Purification via preparative RP-HPLC, isocratic with 14% methanol, yielded a white solid; Yield: 118.1 mg (43%); ^{1}H-NMR (DMSO-d_6): δ = 9.84–9.42 (m, 1H, NH_2), 8.20 (bs, 1H, NH_2), 7.70 (bs, 1H, NH_2), 6.98 (bs, 1H, NH_2), 6.43 (t, J = 5.9 Hz, 1H, NH), 3.51–3.41 (m, 1H, NCH_2), 3.35–3.25 (m, 1H, NCH_2), 3.13–2.94 (m, 2H, NHCH_2), 2.62–2.55 (m, 1H, CH_{dach}), 2.58 (s, 4H, COCH_2), 2.54–2.46 (m, 1H, CH_{dach}), 2.41 (s, 3H, CH_3), 2.17–2.08 (m, 1H, C$H_{2,dach}$), 2.07–1.96 (m, 1H, C$H_{2,dach}$), 1.58–1.42 (m, 3H, C$H_{2,dach}$), 1.41–1.27 (m, 1H, C$H_{2,dach}$), 1.22–1.08 (m, 2H, C$H_{2,dach}$) ppm; ^{13}C-NMR (DMSO-d_6): δ = 177.7 (COCH_2), 165.3 (CONH), 163.7 (CO_{ox}), 163.6 (CO_{ox}), 61.3 (CH_{dach}), 59.5 (CH_{dach}), 57.4 (CH_3), 38.2 (NHCH_2), 38.1 (NCH_2), 30.6 ($CH_{2,dach}$), 28.0 ($CH_{2,dach}$), 23.7 ($CH_{2,dach}$), 23.6 ($CH_{2,dach}$) ppm; ^{195}Pt-NMR (DMSO-d_6): δ = 3000 (major), 2992 (minor) ppm; HRMS (ESI-TOF): calcd. for $[C_{16}H_{26}N_4O_9Pt\text{-}Na^+]^+$: 636.1245, found: 636.1249; elemental analysis calcd. for $C_{16}H_{26}N_4O_9Pt{\cdot}H_2O$: C: 30:43, H: 4:47, N: 8:87, found: C: 30:26, H: 4:59, N: 8:72

Compound 14

(*OC*-6-34)-Acetato[(1*R*,2*R*)-cyclohexane-1,2-diamine]oxalato[2-(2,5-dioxopyrrolidin-1-yl)ethyl)carbamato]platinum(IV)

The compound was synthesized from (*OC*-6-44)-acetato[(1*R*,2*R*)-cyclohexane-1,2-diamine]hydroxidooxalatoplatinum(IV) (200 mg, 0.42 mmol) and 1-(2-

isocyanatoethyl)pyrrolidine-2,5-dione (1.5 eq., 107 mg, 0.63 mmol) in 4 mL DMF (abs.). Purification via preparative RP-HPLC, isocratic with 17% methanol, yielded a white solid; Yield: 92.2 mg (18%); ^{1}H-NMR (DMSO-d_6): δ = 9.76–9.25 (m, 1H, NH_2), 8.74–8.02 (m, 3H, NH_2), 6.76 (t, J = 6.0 Hz, 1H, NH), 3.53–3.42 (m, 1H, NCH_2), 3.37–3.25 (m, 1H, NCH_2), 3.14–2.95 (m, 2H, NHCH_2), 2.71–2.62 (m, 1H, CH_{dach}), 2.62–2.53 (m, 1H, CH_{dach}), 2.58 (s, 4H, COCH_2), 2.14 (bs, 2H, C$H_{2,dach}$), 1.95 (s, 1H, CH_3), 1.57–1.33 (m, 4H, C$H_{2,dach}$), 1.29–1.10 (m, 2H, C$H_{2,dach}$) ppm; ^{13}C-NMR (DMSO-d_6): δ = 178.4 (COCH$_3$), 177.7 (COCH), 164.2 (CONH), 163.3 (CO_{ox}), 61.1 (CH$_{dach}$), 60.8 (CH$_{dach}$), 38.1 (NHCH$_2$), 37.9 (NCH$_2$), 31.0 (CH$_{2,dach}$), 30.9 (CH$_{2,dach}$), 28.0 (COCH$_2$), 23.5 (CH$_{2,dach}$), 23.4 (CH$_{2,dach}$), 22.9 (CH$_3$) ppm; ^{195}Pt-NMR (DMSO-d_6): δ = 3236 (major), 3225 (minor) ppm; HRMS (ESI-TOF): calcd. for $[C_{17}H_{26}N_4O_{10}Pt\text{-}Na^+]^+$: 664.1194, found: 664.1189; elemental analysis calcd. for $C_{17}H_{26}N_4O_{10}Pt\cdot1.5H_2O$: C: 30.54, H: 4.37, N: 8.38, found: C: 30.37, H: 3.98, N: 8.24

Single crystal X-ray crystallography

The X-ray intensity data were measured on a Bruker D8-Venture equipped with a multilayer monochromator, a Cu-K_{α} INCOATEC micro focus sealed tube and Kryoflex II cooling device. The results were uploaded to the CCDC (codes see Table S1). The structures were solved by direct methods and refined by full-matrix least-squares techniques. Non-hydrogen atoms were refined with anisotropic displacement parameters. Hydrogen atoms were inserted in calculated positions and refined with a riding model respectively as rotating systems. The following software was used: Frame integration, *Bruker SAINT software package*[7] using a narrow-frame algorithm, Absorption correction, *SADABS,*[8] structure solution, *SHELXS-97,*[9] refinement, *SHELXL-2013,*[9] *SHELXLE,*[10] molecular diagrams and *OLEX2*.[11]

Experimental data can be found in Table S1. Crystal data, data collection parameters, and structure refinement details are given in Table S2–S3 and Table S5–S6. Molecular Structures in "Ortep View" are depicted in Figure S1 and Figure S2.

Table S1: Experimental parameters and CCDC-codes.

Sample	Temp [K]	Detector distance [mm]	Time/Frame [s]	#Frames	Frame width [°]	CCDC
**7**	100 (2)	40	40	3724	0.4	1499561
**10**	100 (2)	35	35	2646	0.4	1499562

Compound 7: (*OC*-6-44)-Diamminedichloridomethoxido[2-(2,5-dioxo-2,5-dihydro-1*H*-pyrrol-1-yl)ethylcarbamato]platinum(IV)

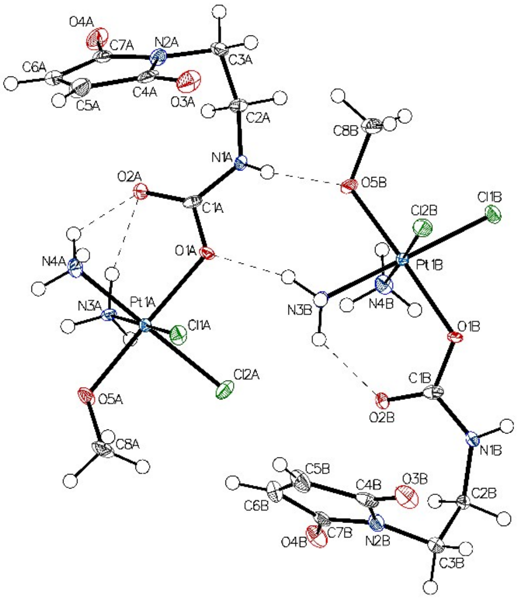

Figure S1: Asymmetric unit of **7** drawn at 50% displacement ellipsoids.

Table S2: Sample and crystal data of **7**.

**Chemical formula**	C8H16Cl2N4O5Pt	**Crystal system**	monoclinic	
**Formula weight [g/mol]**	514.24	**Space group**	*P21/c*	
**Temperature [K]**	100.0	**Z**	8	
**Measurement method**	\Φ and \ω scans	**Volume [Å³]**	2835.2(3)	
**Radiation (wavelength [Å])**	MoKα (λ = 0.71073)	**Unit cell dimensions [Å] and [°]**	12.2584(7)	90
**Crystal size / [mm³]**	0.053 × 0.038 × 0.009		10.9135(6)	104.6597(18)
**Crystal habit**	clear, colorless plate		21.9058(13)	90
**Density (calculated) / [g/cm³]**	2409	**Absorption coefficient / [mm⁻¹]**	10299	
**Abs. correction Tmin**	0.6112	**Abs. correction Tmax**	0.7460	
**Abs. correction type**	multiscan	**F(000) [e⁻]**	1952.0	

Table S3: Data collection and structure refinement of compound **7**.

<table>
<tr><td>Index ranges</td><td>-14 ≤ h ≤ 14, -13 ≤ k ≤ 13, -26 ≤ l ≤ 26</td><td>Theta range for data collection [°]</td><td colspan="2">4.198 to 50.698</td></tr>
<tr><td>Reflections number</td><td>89933</td><td>Data / restraints / parameters</td><td colspan="2">5193/0/375</td></tr>
<tr><td>Refinement method</td><td>Least squares</td><td rowspan="2">Final R indices</td><td>all data</td><td>R1 = 0.0280, wR2 = 0.0443</td></tr>
<tr><td>Function minimized</td><td>Σ w(F_o^2 - F_c^2)^2</td><td>I>2σ(I)</td><td>R1 = 0.0218, wR2 = 0.0429</td></tr>
<tr><td>Goodness-of-fit on F^2</td><td>1148</td><td rowspan="2">Weighting scheme</td><td colspan="2">w=1/[σ^2(F_o^2)+(-0.0134P)^2+9.9013P]</td></tr>
<tr><td>Largest diff. peak and hole [e Å^{-3}]</td><td>1.16/-0.67</td><td colspan="2">where P=(F_o^2+2F_c^2)/3</td></tr>
</table>

Table S4: Selected bond lengths (Å) and angles (°) of **7**.

Pt(1A)-Cl(1A)	2.3132(11)	Pt(1B)-Cl(1B)	2.3012(10)
Pt(1A)-Cl(2A)	2.3248(11)	Pt(1B)-Cl(2B)	2.2951(11)
Pt(1A)-N(3A)	2.044(3)	Pt(1B)-N(3B)	2.043(3)
Pt(1A)-N(4A)	2.030(4)	Pt(1B)-N(4B)	2.058(4)
Pt(1A)-O(1A)	2.040(3)	Pt(1B)-O(1B)	2.049(3)
Pt(1A)-O(5A)	1.980(3)	Pt(1B)-O(5B)	1.978(3)
Cl(1A)-Pt(1A)-Cl(2A)	91.44(4)	Cl(2B)-Pt(1B)-Cl(1B)	91.76(4)
N(4A)-Pt(1A)-N(3A)	85.92(14)	N(3B)-Pt(1B)-N(4B)	91.29(15)
O(5A)-Pt(1A)-O(1A)	178.67(13)	O(5B)-Pt(1B)-O(1B)	178.07(12)

Compound 10: (*OC*-6-44)-Acetatodiamminedichlorido[2-(2,5-dioxopyrrolidin-1-yl)ethyl)carbamato]platinum(IV)

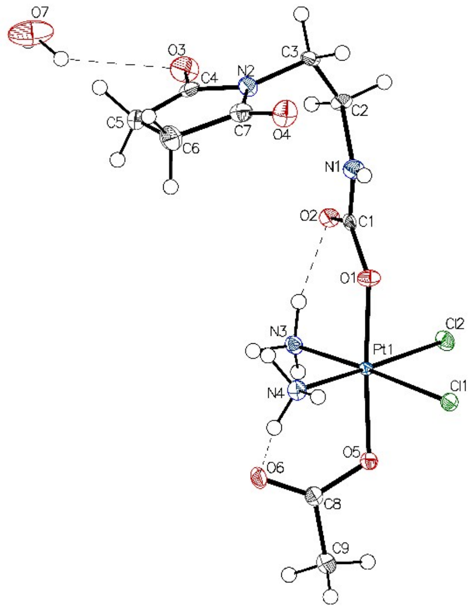

Figure S2: Asymmetric unit of **10**, drawn at 50% displacement ellipsoids. Two Platon Alerts Level B (971) are caused by residual densities close to Pt. Also the weighting scheme points out problems in the convergence of physical properties and structure refinement.

Table S5: Sample and crystal data of **10**.

**Chemical formula**	C9H20N4O7Cl2Pt	**Crystal system**	monoclinic	
**Formula weight [g/mol]**	562.28	**Space group**	*C2/c*	
**Temperature [K]**	100.0	**Z**	8	
**Measurement method**	\Φ and \ω scans	**Volume [Å³]**	3365.1(8)	
**Radiation (wavelength [Å])**	MoKα (λ = 0.71073)	**Unit cell dimensions [Å] and [°]**	27.345(5)	90
**Crystal size / [mm³]**	0.258 × 0.212 × 0.108		10.1753(11)	101.328(5)
**Crystal habit**	clear, colorless block		12.3345(13)	90
**Density (calculated) / [g/cm³]**	2220	**Absorption coefficient / [mm^{-1}]**	8696	
**Abs. correction Tmin**	0.3897	**Abs. correction Tmax**	0.7452	
**Abs. correction type**	multiscan	**F(000) [e$^-$]**	2160.0	

Table S6: Data collection and structure refinement of **10**.

**Index ranges**	-32 ≤ h ≤ 32, -12 ≤ k ≤ 12, -14 ≤ l ≤ 14	**Theta range for data collection [°]**	4.282 to 50.694	
**Reflections number**	44872	**Data / restraints / parameters**	3089/0/214	
**Refinement method**	Least squares	**Final R indices**	all data	R1 = 0.0263, wR2 = 0.0670
**Function minimized**	Σ w(F_o^2 - F_c^2)^2		I>2σ(I)	R1 = 0.0241, wR2 = 0.0638
**Goodness-of-fit on F²**	1089	**Weighting scheme**	w=1/[σ^2(F_o^2)+(0.0368P)^2+20.9744P]	
**Largest diff. peak and hole [e Å^{-3}]**	2.37/-1.28		where P=(F_o^2+2F_c^2)/3	

Table S7: Selected bond lengths (Å) and angles (°) of **10**.

Pt(1)-Cl(1)	2.3382(8)
Pt(1)-Cl(2)	2.3216(9)
Pt(1)-N(3)	2.037(3)
Pt(1)-N(4)	2.044(3)
Pt(1)-O(1)	2.015(3)
Pt(1)-O(5)	2.012(3)
Cl(2)-Pt(1)-Cl(1)	91.06(3)
N(3)-Pt(1)-N(4)	92.20(12)
O(5)-Pt(1)-O(1)	175.46(10)

RP-HPLC stability and binding studies

To determine the stability, especially of the maleimide-function, 0.5 mM solutions of the complexes were prepared in triple distilled water and measured repeatedly over several hours. For determination of the binding rate towards human serum albumin (HSA), freshly prepared 1 mM aqueous solutions of a compound and HSA were mixed 1:1 and also measured repeatedly over several hours. All studies were carried out using a reversed-phase Waters XBridge C18 column on a Dionex Summit System. Milli-Q water, containing 0.1% formic acid, and methanol were used as eluents and a standard gradient from 5–95% methanol in 20 minutes was chosen. The autosampler, where the samples were incubated, was temperature controlled at 20°C and the column at 25°C. The loss of the complexes was monitored via UV-VIS detector at 225 nm and evaluated by the decrease of the compound peak area.

SEC-ICP-MS studies

A reagent I grade water (>10 MΩ cm^{-1} resistance according to ISO 3696 water specifications) purification system (Ultra Clear basic Reinstwassersystem, SG Wasseraufbereitung und Regenerierstation GmbH, Barsbüttel, Germany) was used. Ammonium acetate (≥99%), glutathione disulfide (≥98%), human serum albumin (96-99%), myoglobine from equine skeletal muscle (95-100%) and homocysteine (≥98%) were purchased from Sigma Aldrich, St. Louis, MO, USA. The platinum standard (1002±6 µg/mL) was purchased from Inorganic Ventures, Christiansburg, Virginia, USA. Methionine (≥99%) and glutathione (≥99%) were purchased from Merck (Darmstadt, Germany). Fetal Calf Serum (FCS) was kindly provided by Prof. Walter Berger from the Institute of Cancer Research, University of Vienna, Austria.

SEC-ICP-MS stability and FCS binding studies

For sample preparation compounds **7–14** were incubated separately in FCS. For this purpose they were dissolved in water and finally diluted 1:10 in FCS to obtain a final concentration of 100 µM. The samples were then stored in the autosampler at 37°C for 21 h and analyzed every 2:20 h.

For ICP-MS measurements an ICP-QMS ELAN DRC II PerkinElmer (Ontario, Canada) equipped with a dynamic reaction cell was used. Oxygen (purity 4.5, Linde Gas GmbH, Vienna, Austria) was used as reaction gas. The ICP-MS operation parameters are given in Table S8.

Table S8: ICP-MS operation parameters.

Nebulizer:	PFA-ST
Spray chamber:	Cyclonic
Nebulizer gas flow:	0.9 L/min
Aux. gas flow:	1.3 L/min
Plasma gas flow:	16 L/min
Reaction gas (oxygen) flow:	0.8 L/min
ICP RF power:	1300 W
m/z measured :	194.97, 47.97

As HPLC a Rheos System (Rheos 2000, Flux Instruments AG, Basel, Switzerland) in combination with a metal-free autosampler (AS 50 autosampler) and an ICS-3000DP dual pump system– all from Dionex (Sunnyvale, CA, USA) was coupled to the ICP-QMS ELAN DRC II from PerkinElmer. Chromeleon Chromatography Management System (Version 6.40) was used for data analysis.

Table S9: Chromatographic conditions.

HPLC column:	BioBasic SEC-60 A, 4.6x250 mm, 5µm.
Eluent:	50 mM CH_3COONH_4, pH = 6.0.
Flow rate:	250 µL/min
Split flow (entering the ICP-MS):	125 µL/min
Injection volume:	2 µL
Column temperature:	Room temperature
Autosampler temperature:	37°C

The analysis was carried out in isocratic mode and the flow was split 1:1 after the column since the concentration (100 µM) for the incubation carried out in the autosampler was too high and not suitable for the ICP-MS detector. The chromatographic conditions are shown in Table S9.

A flow injection analysis (FI) was carried out to determine the real concentration of the platinum drugs in FCS and the column recovery. The column recovery was determined at time 0 h of incubation. The results are given in Table S10.

Table S10: Column recovery.

Drug	Conc. (µM)	SD	Column Recovery (%)	SD
**7**	112	5	78	4
**8**	122	1	86	1
**9**	98	4	76	5
**10**	115	3	83	1
**11**	105	2	76	6
**12**	111	2	81	1
**13**	126	0	80	1
**14**	134	3	82	1

A protein/amino acid mix was prepared to assess the separation performance of the applied SEC-HPLC setup. The mixture contained HSA, myoglobin, GSSG, and methionine. It has to be mentioned that HSA is forming dimers over time. However, the separation of the monomer and the dimer of HSA cannot be resolved with this chromatographic column.

Table S11: Resulting size ladder.

Compound	Time [min]	kDa
Albumin (HSA)	7.6	66
Myoglobin	7.9	17
GSSG	9.2	0.6
Methionine	13.0	0.1

SEC-ICP-MS measurements of *in vivo* serum samples

The ICP-QMS iCAP Thermo Scientific (Bremen, Germany) was used. Oxygen (purity 4.5, Linde Gas GmbH, Vienna, Austria) was used as reaction gas. The ICP-MS operation parameters are given in Table S12.

Table S12: ICP-MS operation parameters.

Nebulizer:	PFA-ST
Spray chamber:	Cyclonic
Nebulizer gas flow:	1.01 L/min
Aux. gas flow:	0.80 L/min
Plasma gas flow:	14 L/min
Reaction gas (oxygen) flow:	0.370 mL/min
ICP RF power:	1550 W
m/z measured:	194.97, 47.97

As HPLC system a Thermo Scientific Transcend HPLC system (San Jose, CA, USA) was coupled to the ICP-QMS iCAP Thermo Scientific. Qtegra Intelligent Scientific Data Solution (version 2.4.1800.33) was employed for the data treatment. HPLC chromatographic conditions are given in Table S13.

Table S13: Chromatographic conditions.

HPLC column:	BioBasic SEC-60 A, 4.6x250 mm, 5µm.
Eluent:	50 mM CH_3COONH_4, pH = 6.0.
Flow Rate:	250 µL/min
Injection Volume:	10 µL
Column Temperature:	Room temperature
Autosampler Temperature:	37°C

A flow injection analysis (FI) was carried out to determine the real concentration of the platinum drugs in FCS and the column recovery. The results are given in Table S14.

Table S14: Column recovery.

Drug	Mouse	Pt conc. (µg/L)	St. dev.	RSD (%)	Column recovery (%)	St. dev.	RSD (%)
**12**	#1	5590	54	1	95	1	1
	#2	6068	75	1	85	1	1
	#3	6305	43	1	95	1	1
	#4	6304	28	0	42	0	0
**11**	#1	5869	41	1	70	1	1
	#2	6213	65	1	75	1	1
	#3	6546	52	1	84	1	1
	#4	7675	88	1	98	1	1
**8**	#1	3230	36	1	75	1	1
	#2	2506	27	1	78	1	1
	#3	2671	17	1	77	1	1
	#4	2798	25	1	72	1	1
**Cisplatin**	#1	48	1.5	3	88	11	12
	#2	46	0.4	1	99	4	4
	#3	43	0.2	0	80	2	2
	#4	44	1.0	2	94	10	10

DLS measurements

The samples were prepared by incubating 100 µM HSA with either 5 eq. of the oxaliplatin-based maleimide compound **12** or 5 eq. of the cisplatin-based maleimide compound **8** in 50 mM phosphate buffer (pH=7.4) for 6 h on a stirring table. As a reference, a HSA sample in 50 mM phosphate buffer (pH=7.4) without addition of a platinum compound was also put on a stirring table for 6 h. Prior to the measurements, the samples were diluted 1/9 (v/v) with 50 mM phosphate buffer (pH=7.4). The DLS spectra were recorded on a Malvern ZetaSizer Nano ZS (Malvern Instruments Ltd., Malvern, UK) equipped with a 4 mW He–Ne, 632.8 nm laser beam at 25 °C and at a scattering angle of 173 °.

In vivo studies

Cell culture

The murine (Balb/c) colon cancer cell model CT-26 (purchased from American Type Culture Collection, Manassas, VA, USA) was used. Cells were grown in DMEM/F12 (1:1) supplemented with 10% FCS. All cell culture media and reagents were purchased from Sigma-Aldrich Austria.

Animals

Six- to eight-week-old Balb/c mice (~20-25 g) were purchased from Harlan Laboratories (San Pietro al Natisone, Italy). The animals were kept in a pathogen-free environment and every procedure was done in a laminar airflow cabinet. The experiments were done according to the regulations of the Ethics Committee for the Care and Use of Laboratory Animals at the Medical University Vienna (proposal number BMWF-66.009/0084-II/3b/2013), the U.S. Public Health Service Policy on Human Care and Use of Laboratory Animals as well as the United Kingdom Coordinating Committee on Cancer Prevention Research's Guidelines for the Welfare of Animals in Experimental Neoplasia.

CT-26 experiments

CT-26 cells ($5x10^5$ in serum-free medium) were injected subcutaneously into the right flank of Balb/c mice (gender is indicated in the respective figure). Animals were treated with the indicated oxaliplatin-releasing drugs at concentrations equimolar to 9 mg/kg oxaliplatin (i.v.; **12** was dissolved in 0.9% NaCl solution, **11** in 10% propylene glycol/0.9% NaCl, and oxaliplatin in 5% glucose solution according to the clinical protocol) twice a week (Mondays and Thursdays). In case of cisplatin-releasing prodrugs, a dose equimolar to 3 mg/kg cisplatin was applied twice a week. For all experiments four animals per treatment group were used. Animals were controlled for distress development every day and tumor size was assessed regularly by caliper measurement. Tumor volume was calculated using the formula: (length × width^2). The experiments with oxaliplatin, **11** and **12** in male mice were performed twice with two different endpoints: 1) overall survival and 2) collection of organ and tumor material 24 h after the last day of treatment. In the second experiments, tumors were formalin-fixed and paraffin-embedded. The experiment with oxaliplatin and **12** in female

mice was performed only with the endpoint overall survival. For cisplatin and **8**, the experiment with endpoint of organ and tumor collection 24 h after the last day of treatment was performed. For histological evaluation, tumor tissues were sliced and haematoxylin/eosin stained by routine procedures.

Organ distribution experiments

CT-26 cells ($5x10^5$ in serum-free medium) were injected subcutaneously into the right flank of Balb/c mice. When tumors reached a size of about 500 mm^3, animals were treated once with the platinum compounds as described above. After 24 h, animals were anaesthetized and sacrificed by heart puncture. Serum was isolated from the collected blood samples by centrifugation at 3000 rpm for 10 min for two times and stored together with the collected tissue samples at -20 °C.

Statistics

Results were analyzed and illustrated and statistical analyses performed (as indicated) with GraphPad Prism (GraphPad Software version 5,San Diego, CA).

Determination of the platinum concentration in mice tissues by ICP-MS

Digestion of tissue and blood samples (approx. 20 mg or 50 µL gravimetrically) were performed with 2 ml of nitric acid (~30%) using a microwave system (Discover SP-D,CEM Microwave Technology, Germany). The following microwave parameters were used: 200 °C, 5 min ramp time, 6 min hold time and 300 W maximal power. Digested samples were diluted with Milli-Q water resulting in nitric acid concentrations <4% and platinum concentrations <15 µg/kg.

Milli-Q water (18.2 MΩ cm, Milli-Q Advantage, Darmstadt, Germany) was used for all dilutions for ICP-MS measurements. Nitric acid (≥69%, TraceSELECT®, Fluka, Buchs, Switzerland) was used without further purification. Rhenium and platinum standards for ICP-MS measurements were derived from CPI International (Amsterdam, the Netherlands). All other reagents and solvents were obtained from commercial sources and were used without further purification.

The ICP-MS Agilent 7500ce (Agilent Technologies, Waldbronn, Germany) was equipped with a CETAC ASX-520 autosampler (Nebraska, USA) and a MicroMist nebulizer at a sample uptake rate of approx. 0.25 ml/min. The Agilent MassHunter software package (Workstation Software, version B.01.01, Build 123.11, Patch 4, 2012) was used for data processing. The experimental parameters for ICP-MS are summarized in Table S15. The instrument was tuned on daily basis to achieve maximum sensitivity.

Table S15: ICP-MS parameters Agilent 7500ce.

RF power:	1560 W
Cone material:	Nickel
Carrier gas:	0.8–1.2 L/min
Make up gas:	0.1–0.2 L/min
Plasma gas:	15 L/min
Monitored isotopes:	^{185}Re, ^{194}Pt, ^{195}Pt
Dwell time:	0.3 s
Number of replicates:	10
Number of sweeps:	100

Reduction experiments

1 mM solutions of the succinimide derivatives **9**–**10** and **13**–**14** in 100 mM phosphate buffer (pH=7.4) were freshly prepared, mixed with 10 mM solutions of ascorbic acid in 100 mM phosphate buffer (pH=7.4) and measured by RP-HPLC every hour for up to 24 h. The measurements were performed on a Dionex Summit System with a Waters XBridge C18 column. Milli-Q water, containing 0.1% formic acid, and methanol were used as eluents and a standard gradient from 5–95% methanol in 20 minutes was chosen. The autosampler, where the samples were incubated, was temperature controlled at 20°C and the column at 25°C. The reduction of the compound was monitored via UV-VIS detector at 225 nm and evaluated by the decrease of the initial compound peak area. Additionally, 1 mM solutions of the succinimide derivatives **9**–**10** and **13**–**14** in 50 mM phosphate buffer (in D_2O, pD=7.4) were freshly prepared, mixed with 10 mM solutions of ascorbic acid in 50 mM phosphate buffer (in D_2O, pD=7.4) and measured by ^{1}H-NMR spectroscopy at different time points within the first 12–15 h (in case of the cisplatin-based derivatives **9**–**10**) and within the first 42–48 h (in case of the oxaliplatin-based compounds **13**–**14**), respectively.

Platinum release from HSA-adduct

For the platinum release experiment, a 200 µM solution of compound **8** in 100 mM phosphate buffer (pH=7.4) was mixed 1:1 with a 1 mM solution of HSA (5 eq.) in 100 mM phosphate buffer (pH=7.4). The solution was shaken on a stirring table at room temperature for 24 h. After filtration through a 10 cm Sephadex G25 column, the high-molecular weight fraction was incubated with 10 eq. of ascorbic acid at room temperature for another 24 h. The resulting solution was measured by FI-ICP-MS for the total platinum content. Thereafter, the solution was filtered by centrifugation through a 10 kDa Amicon cut-off filter to remove the high-molecular-weight fraction and the resulting solution was analyzed by RP-HPLC chromatography coupled to an ICP-MS instrument. The result was compared to a reference run of a cisplatin standard. All measurements were performed on an HPLC Agilent 1620 infinity Bio-inert and an Agilent Triple Quadrupole ICP-MS Agilent 8800 (Agilent Technologies, Tokyo, Japan) with Agilent MassHunter software package (Work- station Software, Version C.01.03, 2016) for the data treatment. The instrument parameters and the method are summarized in **Table S16**–**S18**.

Table S16: HPLC Agilent 1620 infinity Bio-inert.

HPLC column	synergi 4 µm Fusion-RP 80A, 2x150 mm.
Eluent	A ≡ 0.1% HCOOH, B ≡ MeOH 0.1% HCOOH
Flow rate	200 µL/min
Injection volume	5 µL
Autosampler temperature	2 °C

Table S17: Gradient method.

Time [min]	A [%]	B [%]
0	100	0
2.5	100	0
6.5	70	30
11.5	70	30
11.6	100	0
15.5	100	0

Table S18: Triple Quadrupole ICP-MS Agilent 8800.

Nebulizer	MicroMist
Spray chamber	Scott double-pass
Nebulizer gas flow	1.05 L/min
Plasma gas	15 L/min
ICP RF power	1550 W
m/z measured	195

Tables and figures

Table S19: Albumin-binding half-lives of compounds **7**–**8** and **11**–**12** evaluated by RP-HPLC.

compound	number of eq. of HSA used	$\lambda_{1/2}$ [h]
**7**	1	5.1
**8**	1	6.3
**11**	1	8.5
**12**	1	7.0
**12**	2	4.5

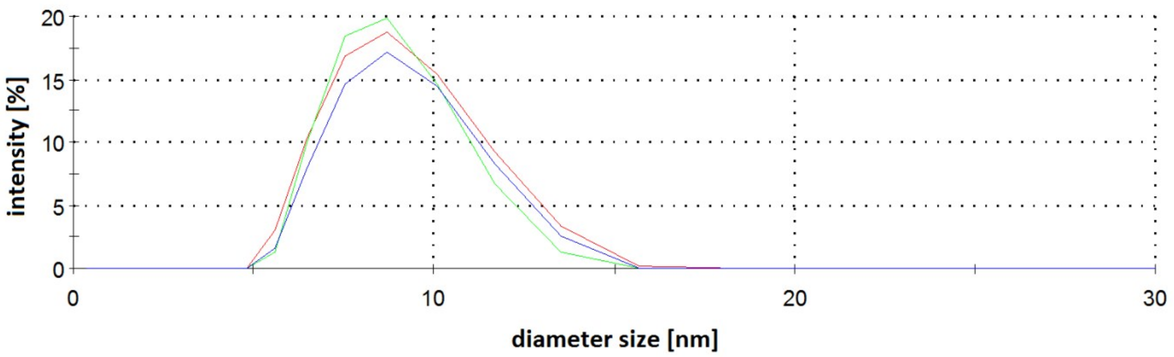

Figure S3: Size determination of HSA (red), HSA incubated with 5 eq. of compound **8** (blue) and HSA incubated with 5 eq. of compound **12** by DLS measurements.

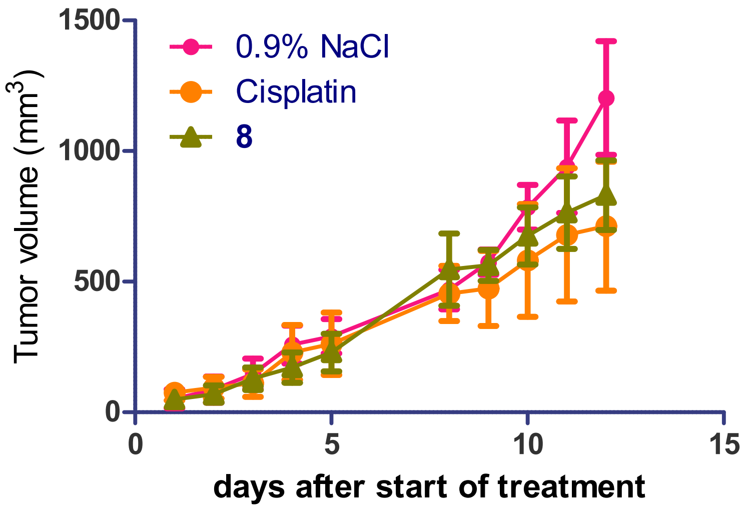

Figure S4: ***In vivo*** **anticancer activity of 8 in comparison to cisplatin:** CT-26 cells were injected subcutaneously into the right flank of male BALB/c mice. Mice were treated twice a week (Monday and Thursday) i.v. with concentrations equimolar to 3 mg/kg cisplatin (each

experimental group contained four animals). Tumor volumes were calculated as described in the experimental part. Data are means ± SEM.

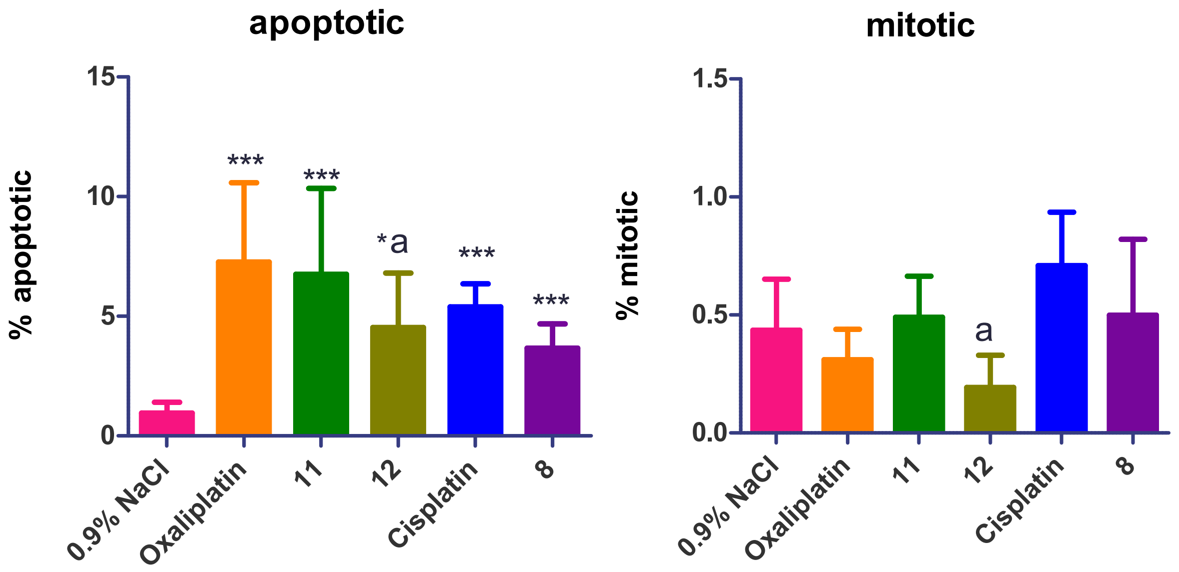

Figure S5: Apoptosis and mitosis levels in tumors after the treatment with compound 8, 11 and 12 in comparison to cis- and oxaliplatin: CT-26 cells were injected subcutaneously into the right flank of male BALB/c mice. Mice were treated twice a week (Monday and Thursday) i.v. with concentrations equimolar to 9 mg/kg oxaliplatin (in case of **11** und **12**) and 3 mg/kg cisplatin (in case of **8**). At day 12 after first treatment, animals were sacrificed and tumor tissues collected, formalin-fixed, paraffin-embedded and H/E-stained. The fractions of apoptotic and mitotic cells were evaluated by counting (4 pictures per sample, at least 300 cells per picture were counted). Each experimental group contained four animals (the respective tumor growth is shown in Figure 4A). [a]n=3 because of one complete remission. **The statistical significance was calculated by One-Way Anova and is indicated with asterisks (* P < 0.05; ** P < 0.01; *** P < 0.001).**

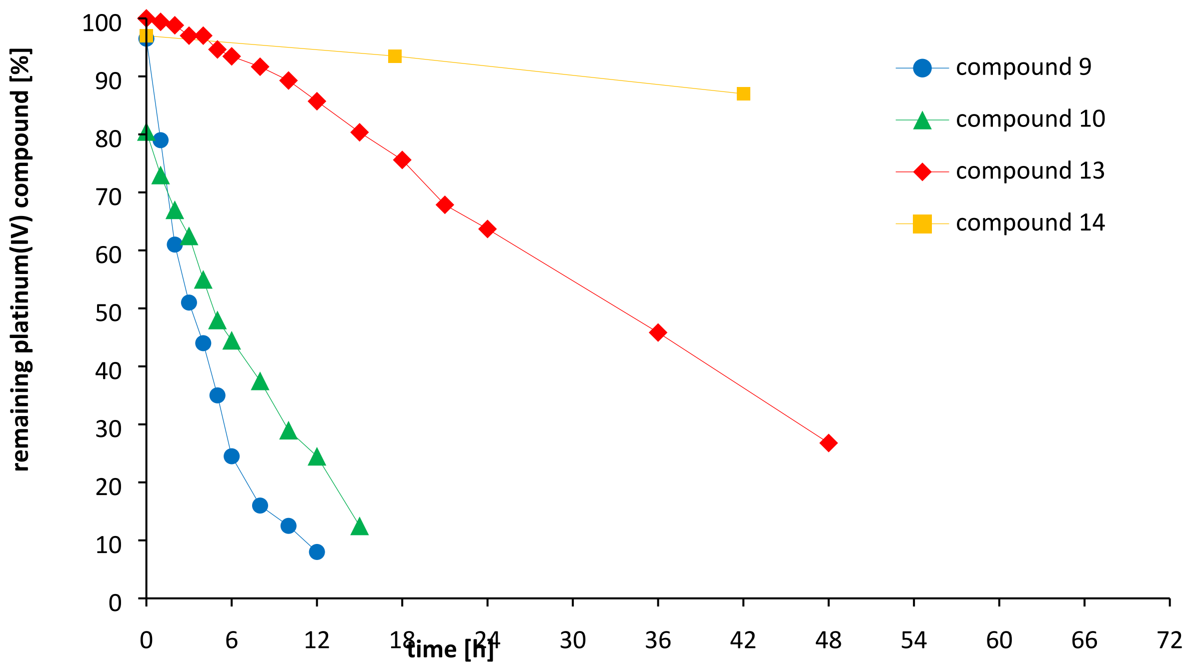

Figure S6: Reduction of the compounds **9–10** and **13–14** with 10 eq. ascorbic acid in phosphate buffer at pH=7.4 monitored by ^{1}H-NMR spectroscopy.

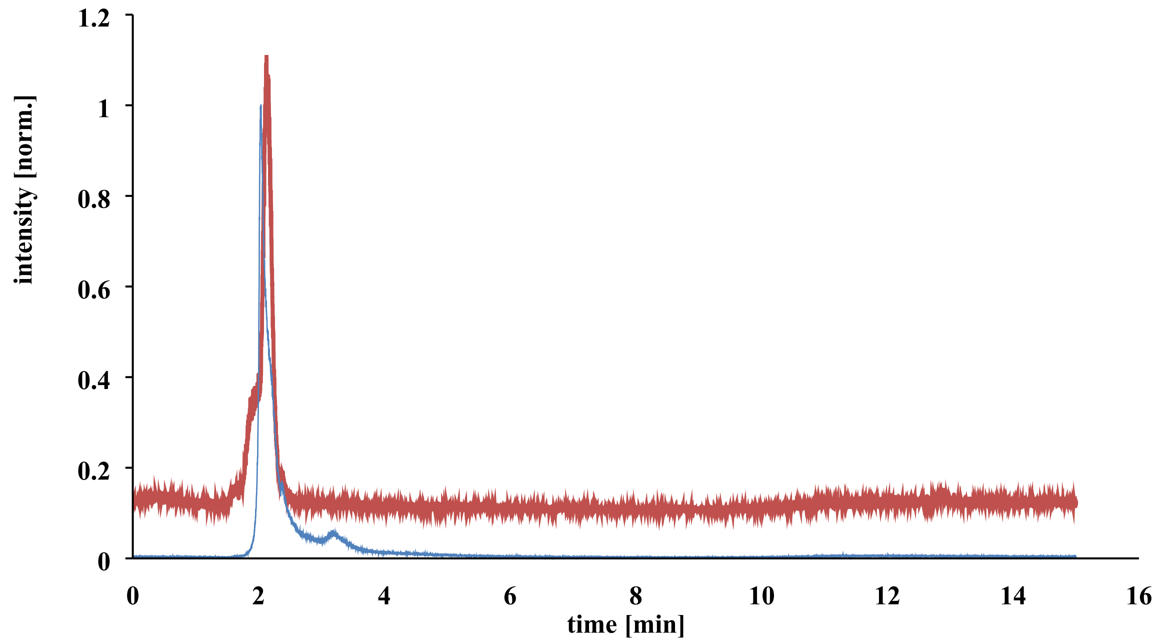

Figure S7: Normalized platinum traces of compound **8** (blue) in comparison to the reference chromatogram of cisplatin (red) measured by RP-HPLC coupled to ICP-MS.

References

1. S. Dhara, *Indian J Chem*, 1970, **8**, 193-194.
2. Y. Kidani, K. Inagaki, M. Iigo, A. Hoshi and K. Kuretani, *J. Med. Chem.*, 1978, **21**, 1315-1318.
3. S. Dunham, R. Larsen and E. Abbott, *Inorg. Chem.*, 1993, **32**, 2049-2055.
4. Y.-a. Lee and O.-s. Jung, *Transition Met. Chem.*, 2004, **29**, 710-713.
5. V. Pichler, J. Mayr, P. Heffeter, O. Domotor, E. A. Enyedy, G. Hermann, D. Groza, G. Kollensperger, M. Galanksi, W. Berger, B. K. Keppler and C. R. Kowol, *Chem. Commun.*, 2013, **49**, 2249-2251.
6. J. J. Wilson and S. J. Lippard, *Inorg. Chem.*, 2011, **50**, 3103-3115.
7. Bruker AXS, *Journal*, 2005-2015.
8. G. M. Sheldrick, *Journal*, 1997.
9. G. M. Sheldrick, *Acta Crystallographica Section A*, 2008, **64**, 112-122.
10. C. B. Hübschle, G. M. Sheldrick and B. Dittrich, *J. Appl. Crystallogr.*, 2011, **44**, 1281-1284.
11. O. V. Dolomanov, L. J. Bourhis, R. J. Gildea, J. A. Howard and H. Puschmann, *J. Appl. Crystallogr.*, 2009, **42**, 339-341.
